# Catalytic oxidation of polystyrene to aromatic oxygenates over a graphitic carbon nitride catalyst

**DOI:** 10.1038/s41467-022-32510-x

**Published:** 2022-08-16

**Authors:** Ruochen Cao, Mei-Qi Zhang, Chaoquan Hu, Dequan Xiao, Meng Wang, Ding Ma

**Affiliations:** 1 Beijing National Laboratory for Molecular Sciences, College of Chemistry and Molecular Engineering, Peking University, Beijing, 100871 People’s Republic of China; 2 State Key Laboratory of Multiphase Complex Systems, Institute of Process Engineering, Chinese Academy of Sciences, Beijing, 100190 People’s Republic of China; 3 Nanjing IPE Institute of Green Manufacturing Industry, Nanjing, 211135 People’s Republic of China; 4 Center for Integrative Materials Discovery, Department of Chemistry and Chemical and Biomedical Engineering, University of New Haven, West Haven, CT 06516 USA

**Keywords:** Heterogeneous catalysis, Photocatalysis, Sustainability

## Abstract

The continuous increase in manufacturing coupled with the difficulty of recycling of plastic products has generated huge amounts of waste plastics. Most of the existing chemical recycling and upcycling methods suffer from harsh conditions and poor product selectivity. Here we demonstrate a photocatalytic method to oxidize polystyrene to aromatic oxygenates under visible light irradiation using heterogeneous graphitic carbon nitride catalysts. Benzoic acid, acetophenone, and benzaldehyde are the dominant products in the liquid phase when the conversion of polystyrene reaches >90% at 150 °C. For the transformation of 0.5 g polystyrene plastic waste, 0.36 g of the aromatic oxygenates is obtained. The reaction mechanism is also investigated with various characterization methods and procedes via polystyrene activation to form hydroxyl and carbonyl groups over its backbone via C–H bond oxidation which is followed by oxidative bond breakage via C–C activation and further oxidation processes to aromatic oxygenates.

## Abstract (web summary)

There is a real need to create materials capable of plastic recycling and upcycling. Here, the authors report a heterogeneous carbon nitride photocatalyst which efficiently converts polystyrene plastics into aromatic oxygenates, such as benzoic acid.

## Introduction

Plastic is an indispensable material with a global production reaching nearly 400 million tons in 2020^1^. In the past, the post-consumer plastics were considered as garbage, and most were landfilled or discarded in the natural environment, leading to serious environmental pollution problems^2–4^. Nowadays, the plastic wastes are considered as an important carbon resource with the development of emerging recovery methods. Chemical recycling is believed to be the most valuable process of all recycling methods^5,6^. Apart from depolymerization into monomers (closed-loop recycling process), researchers have reported that plastic polymers can be transformed into various fuels^7,8^, chemicals^9,10^, and advanced materials^11,12^ via pyrolysis, hydrogenolysis, solvolysis, carbonization, and functionalization, etc. (open-loop recycling processes). In addition to the traditional thermochemical catalytic approaches, photo-^13–15^, electro-^16–18^ catalytic approaches of upcycling plastic wastes have drawn wide attention. Photocatalysis is considered as a green and promising technique. By utilizing energy of light, photocatalytic process can be achieved under mild conditions^13,19^. The different chemical processes of photocatalysis compared with thermal catalysis also make it possible to achieve unique reaction selectivity^14,19,20^. It is particularly attractive to explore the possibility to produce large amount of high-value chemicals from real-life plastic wastes from fundamental knowledge of chemical processes.

Polymers connected by C–C bonds, such as polyethylene (PE), polypropylene (PP), polystyrene (PS) and polyvinyl chloride (PVC), comprise more than 76% of all plastics^21,22^. But they are very inert and usually require pyrolysis or hydrogenolysis methods at high temperatures (typically > 300 °C) for depolymerization. However, products from pyrolysis and hydrogenolysis of these polymers are complex mixtures (except CH_4_^23^) due to random bond scission under harsh conditions. Thus, achieving high selectivity towards one target product from these polymers is another ongoing challenge to produce bulky value-added. Among the C–C bond linked polymers, polystyrene is a special representative because it contains electron-rich conjugated aromatic ring units as functional groups and activated benzylic C–H bonds for selective depolymerization and transformation.

Previous works have exhibited that polystyrene can be selectively converted into the monomer styrene^24^ or other arenes such as benzene^25^ by heterogeneous catalysts, while the upgradation into functionalized aromatics with higher value was mainly achieved by homogeneous catalysts^26^. Very recently, oxidative deconstruction of polystyrene was reported, which depolymerized and transformed the polymer into high-value products with oxygen-containing groups, such as benzoic acid^27–31^. The photocatalytic benzylic C–H bond activation was involved in most of these catalytic processes. For example, it was reported that the radiation generated chlorine radicals from the catalyst FeCl_3_ can extract hydrogen atoms on the PS backbone, and then the activated PS can be oxidatively converted into benzoyl products with a yield up to 23 mol %^27^. Another research describes an acid-catalyzed protocol for polystyrene in which singlet oxygen (^1^O_2_) is demonstrated to be the reactive species to extract hydrogen atoms from tertiary C–H bonds and initiate the hydroperoxidation and C–C bond cleavage^28^. It is well known that heterogeneous photocatalysts can undergo charge separation by irradiation and reactions of photo-generated electrons with O_2_ sequentially form superoxide radicals (•O_2_^−^), peroxide anions (O_2_^2−^), and hydroxyl radicals (•OH). As these reactive oxygen species (•O_2_^−^, O_2_^2−^, or •OH) are highly active towards C–H bond activation, the polystyrene oxidation by heterogeneous photocatalysts is possible and worth exploring.

Heterogeneous catalysts have the advantage of easy to separate, and heterogeneous catalytic systems possess great prospects for the bulk transformation of chemicals. Semiconductor nanomaterials, such as oxides (TiO_2_, ZnO, WO_3_, and ZrO_2_), carbides (SiC and CN_x_) and sulfides (ZnS, MoS_2_, and CdS), are the most commonly used heterogeneous photocatalysts due to their good light absorption properties, chemical stability and tunable nanostructures^14,19,20,32^. Herein, we report a photocatalytic method to oxidize polystyrene to aromatic oxygenates including benzoic acid, acetophenone, and benzaldehyde in liquid phase in the presence of a graphitic carbon nitride (g-C_3_N_4_) catalyst. Excellent reaction performance, i.e., good mass-specific reactivity (about 10 mg·g_catal_^−1^·h^−1^) and high selectivity towards a single high-value chemical (benzoic acid, >80%), is observed at 150 °C under visible light irradiation. The conversion of gram-scale real-life plastic waste is demonstrated. By means of the carefully investigation of recycled polymers during the reaction and control experiments, we conclude that polystyrene undergoes chain oxidation (C–H bond activation) followed by bond scission (C–C bond activation) and further oxidation to mainly benzoic acid via a radical mechanism.

## Results

### Optimization of reaction conditions for photocatalytic oxidation of polystyrene

We firstly choose several heterogeneous photocatalysts (TiO_2_, ZnO, ZnS, and g-C_3_N_4_), which are environmental-friendly, easy to prepare and commonly used in photocatalytic oxidation. When the photocatalytic transformation of commercial polystyrene (weight averaged molecular weight *M*_w_ ~ 50 kDa) was performed under the light irradiation (by a 300 W Xenon lamp) at room temperature in a stainless-steel reactor (1 bar air, acetonitrile as the solvent), no product was observed in 24 h reaction with any catalysts. As we gradually increased the reaction temperature, considerable amounts of products could be observed at 80 °C. We then performed the transformation under the thermal- (80 °C) and light irradiation- (by a 300 W Xenon lamp at 80 °C) treatments with/without heterogeneous photocatalysts (TiO_2_, ZnO, ZnS, and g-C_3_N_4_; 1 bar air; acetonitrile as the solvent). Under light irradiation, aromatic oxygenates including benzoic acid, acetophenone, and benzaldehyde and inorganic products including CO_2_ and CO were obtained on all the tested catalysts (Table 1, entries 2–5). Even without catalyst, certain amounts of organic and inorganic products were detected although the conversion was low (Table 1, entry 1, note that the definition of conversion was different from general definition, see detailed definition in methods section). Instead, no liquid or gas product was detected under thermal catalytic reaction condition (Table 1, entry 6). The conversions of polystyrene were 13%, 21%, 12%, and 46% on TiO_2_, ZnO, ZnS, and g-C_3_N_4_, respectively, with the selectivities of 15%, 55%, 64%, and 60%, respectively. Surely, over the TiO_2_ catalyst, the major products were CO_2_ and CO, suggesting that TiO_2_ is not a good photocatalyst for the conversation of PS. Although good selectivity to the aromatic compounds (>50%) were observed from the ZnO or ZnS catalyst, the overall reactivity was not as high as that of g-C_3_N_4_. Therefore, we choose the g-C_3_N_4_ catalyst, prepared by the thermal condensation of urea^33–35^ with graphitic sheet structure (Supplementary Fig. 1, XRD profiles) and high surface area (33 m^2^·g^−1^ BET surface area, Supplementary Fig. 2), for further investigation. The as-prepared g-C_3_N_4_ catalyst was also characterized by Fourier transform infrared (FTIR) spectroscopy (Supplementary Fig. 3), Ultraviolet and visible (UV-vis) spectroscopy (Supplementary Fig. 4), X-ray photoelectron spectroscopy (XPS, Supplementary Fig. 5) and Raman spectroscopy (Supplementary Fig. 6). These characterization results were consistent with the properties of g-C_3_N_4_ materials reported by the previous literatures^33,36–42^.

**Table 1:** Catalytic performance of polystyrene photo-oxidation with different catalysts and solvents^a^


Entry	Catalyst	Solvent	Temperature (°C)	Gas in the reactor	Conversion^b^ (%)	Selectivity to organics^c^ (%)
1	none	acetonitrile	80	1 bar air	4 ± 1	47 ± 3
2	TiO_2_	acetonitrile	80	1 bar air	13 ± 1	13 ± 1
3	ZnO	acetonitrile	80	1 bar air	21 ± 2	55 ± 3
4	ZnS	acetonitrile	80	1 bar air	12 ± 1	64 ± 4
5	C_3_N_4_	acetonitrile	80	1 bar air	46 ± 3	61 ± 3
6	C_3_N_4_, no light	acetonitrile	80	1 bar air	not detected	–
7	C_3_N_4_	acetone	80	1 bar air	17 ± 1	26 ± 2
8	C_3_N_4_	THF	80	1 bar air	6 ± 1	11 ± 1
9	C_3_N_4_	free	150	1 bar air	10 ± 1	43 ± 2
10^d^	C_3_N_4_	acetonitrile	150	10 bar O_2_	96 ± 6	60 ± 4
11^d,e^	C_3_N_4_	acetonitrile	150	10 bar O_2_	91 ± 6	61 ± 5

^a^Reaction condition: catalyst 50 mg, PS (*M*_w_ ~ 50 kDa) 20 mg, 30 mL solvent in 100 mL autoclave, 300 rpm magnetic stirring, Xenon lamp, 24 h. Errors in this table are standard deviation in 3 parallel experiments.

^b^Conversion (%) = (*n*(COx) + *n*(carbon in benzoic acid, acetophenone and benzaldehyde))/(*n*(carbon in (C_8_H_8_)_*n*_) × 100%. COx produced from solvent oxidation was subtracted, which was estimated by blank test (Supplementary Table 1).

^c^Selectivity (%) = (*n*(carbon in benzoic acid, acetophenone and benzaldehyde)/(*n*(carbon in benzoic acid, acetophenone and benzaldehyde) + *n*(CO_x total_))) × 100%, CO_x_ from solvent oxidation is included.

^d^Optimized reaction condition: catalyst 50 mg, PS (*M*_*w*_ ~ 50kDa) 10 mg, 150 °C, 10 bar O_2_, 30 mL solvent in 100 mL autoclave, 300 rpm magnetic stirring, Xenon lamp, 24 h. Detailed distribution of different species can be seen in Fig. 1a.

^e^Visible light source was used by passing the Xenon light source through a 400–800 nm filter.

Considering that the reaction temperature, pressure, solvent, and metal additives on g-C_3_N_4_ could influence the generation and consumption of reactive oxygen species from the activation of O_2_ and affect the solubility of polystyrene, the mass/heat transfer, the photocatalytic oxidation process has been systematically optimized under different conditions. First, reactions with conventional solvents were evaluated (Table 1, entries 5 and 7–9). Compared to acetonitrile, lower reactivity and selectivity were observed for the reactions in acetone, tetrahydrofuran (THF), and solvent-free system. This may be due to the self-oxidation of acetone and THF that consumes oxygen radicals to compete with the oxidative depolymerization of polystyrene (Supplementary Table 1). Second, the production rate of organic products increased monotonically from 0.1 to 0.9 mmol_carbon_·g_catal_^−1^·h^−1^ with increasing reaction temperature from 80 to 180 °C under low conversions (<20%, Supplementary Fig. 8a, b), while the selectivities (organics vs. CO_x_) were almost unchanged. When high conversions (>80%) were achieved by extending the reaction time, raising the reaction temperature resulted in a significant increase in CO_x_ generation (Supplementary Fig. 8b, c), which may be attributed to the quicker oxidation of the organic products at higher temperatures. Third, increasing the O_2_ pressure (from air to 10 bar O_2_) enhanced the production rate of organic products from 0.45 to 0.90 mmol_carbon_·g_catal_^−1^·h^−1^ at 150 °C in 5 h while the selectivity was maintained (Supplementary Fig. 9). Obviously, the reaction could not happen under a pure N_2_ atmosphere (Supplementary Fig. 9). Lastly, since metal species were reported to enhance the photocatalytic activity for g-C_3_N_4_ catalysts by modifying the band gap of photocatalyst, improving the separation efficiency of the photo-generated electron-hole pairs, promoting the adsorption of the reactants^43^, different metal additives were evaluated. In this case, although the metal modification slightly increased the overall conversion, the selectivity to the target products (aromatic oxygenates) was significantly decreased (Supplementary Table 2), which could result from the over-oxidation of the intermediates/products promoted by the metal additives. In the subsequent experiments, we use pure g-C_3_N_4_ catalyst for further research.

We further extended the photo irradiation time to 24 h (10 bar of O_2_, pure g-C_3_N_4_ catalyst and at 150 °C), and the selectivity to the organics was still 59% while almost unit of conversion was achieved (Table 1, entry 10). When visible light was used (400–800 nm), the catalytic performance remained nearly the same (Table 1, entry 11). By applying the optimized reaction conditions to the polystyrene with different molecular weight (*M*_w_ ~ 800 Da, 2500 Da, 12 kDa, 50 kDa and 110 kDa), syndiotactic PS and real-life PS-containing plastics (e.g., pellets and cups), similar productivities and selectivities were obtained (Supplementary Table 3). The polystyrene with lower molecular weights showed slightly higher reaction activities, which may be due to the good solubility by the solvent. In addition, excellent reusability of g-C_3_N_4_ was confirmed by testing the recovered catalyst for five runs, where the reactivity and selectivity of the reactions were well maintained (Supplementary Fig. 10).

### Study on the mechanism of photocatalytic oxidation of polystyrene

**Fig. 1:**
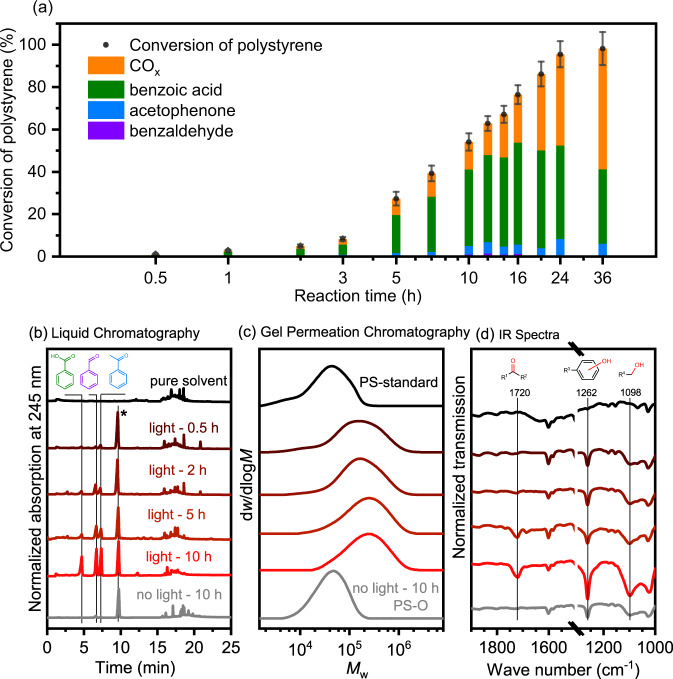
Analysis of product and PS reactant in different reaction stages. **a** Time-evolution of different products for polystyrene photocatalytic oxidation reaction at 150 °C under 10 bar O_2_, 50 mg g-C_3_N_4_, 10 mg polystyrene (*M*_w_ ~ 50 kDa), 30 mL acetonitrile, 300 W Xenon lamp. Distribution of different products are shown by column with different colors. The error bars represent the standard deviation of conversion in 3 parallel experiments. **b** Liquid chromatograms of reaction solutions with different time and conditions. The first line pure solvent refers to the signal of pure solvent. The peaks marked with an asterisk represent the nitrobenzene internal standard. **c** Molecular weight distribution measured by Gel Permeation Chromatography (GPC) and **d** the Infrared (IR) transmission spectra of the polystyrene reactant and the recovered polystyrene after reactions with light irradiation for 0.5 h, 2 h, 5 h, and 10 h and without light irradiation for 10 h. The corresponding spectral order in **d** is consistent with that in **c**. Source data are provided as a Source Data file.

We studied production of organic and inorganic products in time-evolution experiments (Fig. 1a, b) and properties of the reacted polystyrenes recovered from different reaction times and conditions (Fig. 1c, d). To reach high conversion within an appropriate time scale, the starting amount of polystyrene substrate has been reduced by half. An induction period was observed in the first 3 h, and the products rapidly accumulated from 3 to 24 h (Fig. 1a and Supplementary Fig. 11a). A small amount of benzoic acid, acetophenone, and benzaldehyde were detected while no dimer, trimer or oligomers of polystyrene were identified in significant amount in the induction period by liquid chromatography (Fig. 1b and Supplementary Fig. 12) and gas chromatography- mass spectrometer (GC-MS, Supplementary Fig. 13). Meanwhile, for the solid in the reaction mixture, instead of depolymerizing to polystyrene intermediates with shorter chains (smaller *M*_w_), the residue polystyrene showed an increase of molecular weight in the first 30 min, and the molecular weight further increased with the extension of reaction time (Fig. 1c). It is interesting to see the increase of molecular weight for recovered polystyrene in the induction period. Here are two possible causes: (i) the introduction of oxygen-functional groups on its lattice, which will be further discussed in following section and (ii) the re-polymerization of polystyrene induced by radical attack^44,45^. But the PS was eventually decomposed into small organic molecules with prolonged reaction time, as even the polystyrene with high molecular weight could be easily converted (Supplementary Table 3). When the target products were quickly produced in 3–10 h, oligomers, dimers or corresponding derivatives (Fig. 1b and Supplementary Figs. 12 and  13) or the decrement on *M*_*w*_ of recovered polystyrene (Fig. 1c) were not detected. This observation indicated that polystyrene was not depolymerized into oligomers as intermediates in the induction period, and instead, something must happen in this circumstance.

The Infrared (IR) absorption measurement of recovered polystyrene samples shed light on the possible reaction pathways. As shown in Fig. 1d, IR transmission spectra of the recovered polystyrene at the early stage of photocatalysis (before 2 h) clearly showed the appearance of two new absorption peaks at 1262 and 1096 cm^−1^, although the rate of products formation was low at this time (Fig. 1a). They are attributed to the C–O bond of phenolic hydroxyl groups and C–O bond of alcoholic hydroxyl, respectively^46^. Later on (2–10 h), a peak raised at 1720 cm^−1^, which can be attributed to the absorption of carbonyl groups (C=O bonds). The signals of phenolic (1262 cm^−1^) and alcoholic (1096 cm^−1^) hydroxyl groups in polymers continued to increase. In addition, the element analysis of recovered polystyrene confirmed that a small content of oxygen was introduced into the polystyrenes (Supplementary Table 4). These findings demonstrated that our heterogonous catalytic transformation process of polystyrene is different from traditional pyrolysis^8,47^ and oxidation^29,31^ reaction routes of polymers, where random C–C bonds were cleaved to produce microplastics or oligomers. Instead, the spectroscopic result together with gel permeation chromatography (GPC) and liquid product analysis clearly indicated that partial oxidation of polystyrene by reactive oxygen species occurred in the induction period of reaction.

**Fig. 2:**
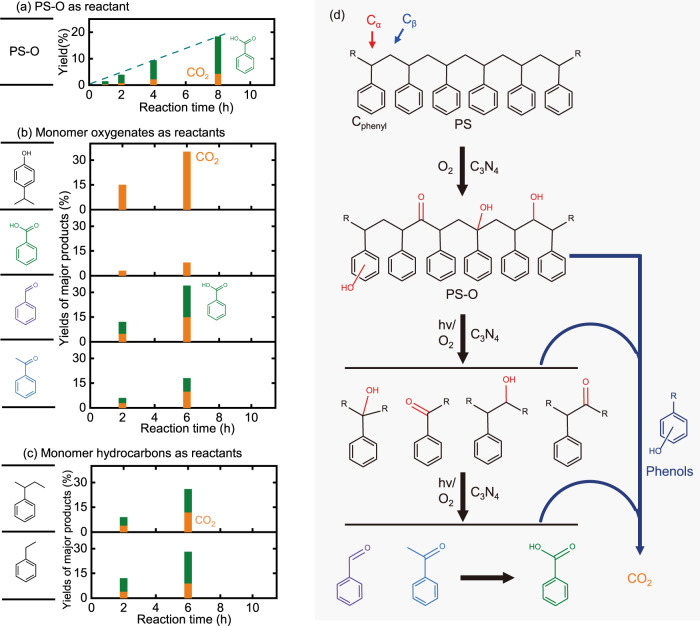
Reaction performances of different model molecules and proposed reaction mechanism. **a**–**c** Catalytic performances of **a** oxygen treated polystyrene, (**b**) oxygenate monomers, and **c** hydrocarbon monomers as reactants under standard reaction conditions. Reaction conditions: 50 mg g-C_3_N_4_, 20 mg reactant, 30 mL acetonitrile, 10 bar O_2_, irradiated by 300 W Xenon lamp at 150 °C. **d** Proposed reaction pathway of polystyrene photo-oxidation reaction. Source data are provided as a Source Data file.

Therefore, it can be assumed that the partial oxidation of aromatic rings and aliphatic carbon chains of polystyrene corresponds to the induction period of the reaction. The partial oxidation (C–H bond activation) seemed easier than the final depolymerization (C–C bond breakage) because almost no products in the liquid and gas phases were obtained after 40 h reaction in dark (150 °C, with catalyst, Supplementary Fig. 11b) while the oxygen containing functional groups were clearly discovered from the corresponding recovered polystyrene (marked as PS-O, Fig. 1d and Supplementary Table 4). Then the PS-O obtained from the partial oxidation with O_2_ under dark condition was exposed under light irradiation. Significantly, the production of aromatic oxygenates from PS-O was observed almost immediately after light on and increased linearly with reaction time, indicating that the induction period was successfully eliminated (Fig. 2a and Supplementary Fig. 11c). This again suggests that the induction period is from the partial oxidation of polystyrene, happened both in dark or under light, which will introduce alcoholic and phenolic and later carbonyl groups over the lattice of polystyrene.

The partially oxidized polystyrene is apt to be attacked by the photo-generated reactive oxygen radicals (Fig. 2d). By using 5,5-dimethyl-1-pyrroline N-oxide (DMPO) as radical capture agents, we used the EPR spectra to study the existence of oxygen-related radicals under the reaction conditions with/without g-C_3_N_4_ catalyst in presence/absence of light at room temperature^48^. Indeed, it was demonstrated that the formation of •O_2_^−^ radicals can only be generated under light irradiation in the presence of g-C_3_N_4_, indicating the importance of both light irradiation and photocatalyst for the generation of active oxygen radical species (Supplementary Fig. 14). We believe that those active oxygen radical species are responsible for the C−C bond breakage in the partially oxidized polystyrene, leading to the formation of small molecular products or intermediates.

Then the possible small molecular products include p-isopropylphenol (as a representative of phenols), benzoic acid, benzaldehyde, acetophenone, ethylbenzene, and sec-butylbenzene were evaluated separately as reactants under typical reaction conditions. They have different reactivity and different products may form under light irradiation/catalyst. As shown in Fig. 2b and Supplementary Fig. 15b, the phenolic compounds were rapidly converted into CO_2_ without considerable amounts of organic products. It explained well that phenols were not obtained as the final products from polystyrene photocatalytic oxidation even though phenolic hydroxyl groups were found from the partially oxidized polystyrene. In contrast to the oxidation of benzoic acid that slowly produced CO_2_ only, benzaldehyde, acetophenone, ethylbenzene, and sec-butylbenzene showed much better reactivity, with benzoic acid and CO_2_ as main products (Fig. 2b, c). Although these molecules might be the potential intermediates from the reaction step of the depolymerization of the partially oxidized polystyrene, they can be easily further oxidized to produce benzoic acid and CO_2_ under the effect of g-C_3_N_4_ catalyst and light irradiation.

A possible mechanism for the oxidative depolymerization of polystyrene was schematically shown in Fig. 2d. In short, the activated intermediate, PS-O, was produced from the oxidative functionalization of polystyrene with OH groups at C_α_ or C_phenyl_ sites as well as OH or C=O groups at C_β_ sites, which can occur under both thermo and photo irradiation conditions in the presence of g-C_3_N_4_. Under light irradiation, photo-generated electrons and holes by g-C_3_N_4_ react with O_2_ and polystyrene respectively, to produce •O_2_^−^ and possible carbon radical intermediates^49^. Then the partially oxidized polystyrene is easily attacked by the reactive oxygen radicals or oxidative photo-generated holes under the effect of g-C_3_N_4_ catalyst and light irradiation to form C−C−O• intermediate, leading to the breakage of C−C bond and scission of polymer backbone by the β-scission process^27,28^. This oxidation and β-scission process was repeated, and finally various small molecular products were produced. The produced intermediates, including p-isopropylphenol, ethylbenzene, and sec-butylbenzene like molecules, can be further oxidized under photocatalysis condition producing benzaldehyde, acetophenone, benzoic acid and CO_2_ as the final products (Fig. 2d). Surely, overoxidation of the carbon containing reactants/intermediates/products could happen in any steps above, resulting the formation of undesired CO_2_ in mass selectivity.

### Further improvement based on the reaction mechanism

In the above discussion, we hypothesized that the following strategies may be helpful to achieve better reaction efficiency and selectivity of value-added organic chemicals from waste polystyrene: (1) pretreat the raw polystyrene via designed processes to obtain the critical intermediates with proper modification; (2) construct a circulatory system to remove the products to prevent the over-oxidation; (3) manipulate the product selectivity by controlling the reaction conditions and the pretreatment of polystyrene; (4) optimize the solvent system to gain a best compromise between the dispersion of polystyrene in the solution and the consumption of reactive oxygen species via self-oxidation of the solvent; (5) explore the photocatalyst systematically to achieve better efficiency. Based on the above strategies, we made some attempts to further optimize the process of organic chemicals synthesis from waste polystyrene.

**Fig. 3:**
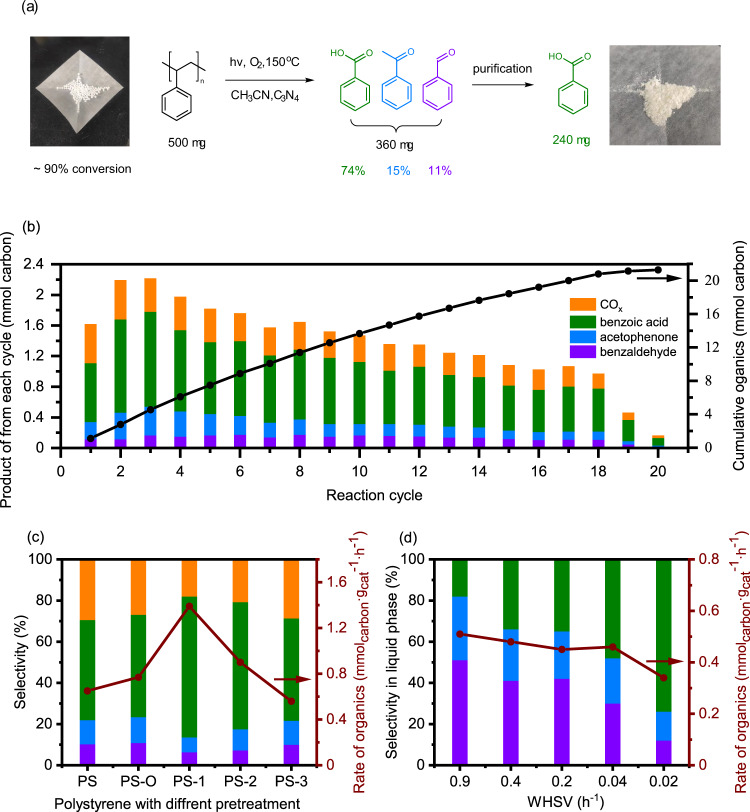
Improvement of reaction performances based on the reaction mechanism. **a** Schematic diagram of the conversion experiment of 500 mg real-life PS pellets; (**b**) Conversion of 500 mg plastic pellets in 20 reaction cycles, reaction conditions: 200 mg g-C_3_N_4_, 500 mg pellets, 40 mL acetonitrile, 10 bar O_2_, irradiated by 300 W Xenon lamp at 150 °C for 8 h in each cycle. The solution is released after each cycle and pure solvent is injected into the autoclave. **c** Catalytic performance of polystyrene with different pretreatment, reaction conditions: 50 mg g-C_3_N_4_, 20 mg polystyrene, 30 mL acetonitrile, irradiated by 300 W Xenon lamp at 150 °C for 8 h. PS-O was obtained from thermal treatment at 150 °C in acetonitrile with O_2_, PS−1 and PS-2 were obtained from air treatment at 220 °C and 300 °C respectively, PS-3 was obtained from pyrolysis at 350 °C in N_2_. **d** Selectivity and activity of polystyrene oxidation reaction at different WHSV, reaction conditions: 100 mg g-C_3_N_4_, 30 mL acetonitrile, irradiated by 300 W Xenon lamp at 120 °C, polystyrene solution (~0.3 mg/mL in acetonitrile) was pumped into the reactor by a high-pressure syringe pump at different rate, 10 mL reaction solution was drained out manually when the PS solution pumped in reached the same volume. The error bars represent the standard deviation of conversion in 3 parallel experiments. Source data are provided as a Source Data file.

Firstly, a circulatory system has been constructed to prevent the overoxidation of benzoic acid and other oxygenates (Supplementary Fig. 7). In each cycle, the insoluble polystyrene and oxygen were kept with the catalyst in the reactor while the solution was drained out through a filter, after that, solvent in equal volume was replenished by an injection pump. With proper control of the substrate/catalyst ratio (5:2) and reaction time (8 h), a steady production rate of the organics (10 mg·g_catal_^−1^·h^−1^) with 76% selectivity was obtained in 18 cycles for the conversion of 0.5 g of plastic pellets, then it rapidly decreased because the polystyrene was almost fully converted (Fig. 3b). We noted that a minority of the pellets were unreacted because they were dissolved in the solvent and taken out from cycling, which could be easily recycled. Eventually, 90% of the plastic pellets were converted and a total amount of 360 mg aromatic oxygenates were produced (74% benzoic acid, 15% acetophenone and 11% benzaldehyde). In addition, pure chemicals can be easily obtained after simple separation, e.g., 240 mg of benzoic acid was recovered from column chromatography separation (Fig. 3a).

Secondly, the pretreatment of polystyrene was explored because it can be easily oxidized or pyrolyzed by thermal treatment to form partially oxidized or relatively soluble precursors respectively. Except the PS-O obtained from 40 h treatment in previous thermal oxidation at 150 °C, three different PS precursors, PS-1, PS-2, and PS-3, were obtained from oxidation in air at 220 °C, 300 °C, and pyrolysis in N_2_ at 350 °C, respectively. The oxidized (PS-O, PS-1, and PS-2) and better dissolved (PS-3) polystyrenes were evaluated in photocatalytic reaction (with g-C_3_N_4_ as catalyst) and compared with pristine material (Fig. 3c). Significant improvements were obtained from PS-1 (220 °C treatment in air) that the reaction rate was doubled comparing to the raw polystyrene and the selectivity to benzoic acid in organics reached 80%. This might be due to the proper partial oxidation of the aliphatic chains. Although advantages on reaction performance from the pyrolysis precursor was not observed, the better solubility of the pretreated polystyrene made the continuous flow reaction system possible.

Therefore, by adapting the soluble polystyrene (>0.3 mg/ml) from partial pyrolysis as reactant, the reaction performance was tested with different weight hourly space velocity (WHSV) of polystyrene in a flow reaction system to optimize the selectivity and activity to specific products. Under high WHSV (0.9 h^−1^), benzaldehyde and acetophenone could be obtained with a higher selectivity (51% and 31%, respectively) in liquid phase. As WHSV decreased, selectivity of benzoic acid gradually increased from 18% to 74%. At a WHSV of 0.02 h^−1^, the selectivity of three products is similar with that of the aforementioned batch reactor. These results verified that it is possible to establish the continuous-flow heterogenous catalysis reaction system for polystyrene oxidation, and the selectivity of oxidation reaction could be manipulated by controlling of reaction conditions. This offers a starting point for further optimization and scale-up of the process.

## Discussion

In this manuscript, a photocatalytic oxidative method was successfully developed for polystyrene conversion to aromatic oxygenates in gram scale by using a heterogeneous g-C_3_N_4_ catalyst. The polystyrene undergoes chain oxidation primarily, and the oxidized polymer gets cleaved and further oxidized to yield small aromatic oxygenates. As a result, the highly efficient polystyrene transformation process with the yield of 60% towards organic products is reached. High selectivity in liquid phase towards single product (74% for benzoic acid or 51% for benzaldehyde in independent experiments) was achieved by tuning WHSV. We demonstrated that this free radical driven catalytic system has good stability and universality for diverse substrates. This heterogeneous catalytic system provides a strategy to upcycle plastic wastes to valuable chemicals with high selectivity.

## Methods

### Catalyst preparation

Graphitic carbon nitride (g-C_3_N_4_) catalysts were prepared by thermal condensation of urea. Typically, 10 g urea was placed in crucible and heated to 550 °C for 4 h in air at a ramp rate of 2 °C/min. The resulting yellow product was grounded into powder and washed by ethanol and water respectively. Yellow powder of g-C_3_N_4_ (about 2 g) was obtained after drying the washed powder at 120 °C over 6 h.

Metal-supported g-C_3_N_4_ catalysts were prepared by wet impregnation method. The as-prepared g-C_3_N_4_ was soaked into deionized water. The water solution of metal precursor was dropped into the slurry of the support to achieve the desired metal loading. The precursors for Au, Pt, Fe and Cu are HAuCl_4_·4H_2_O, H_2_PtCl_6_·6H_2_O, Fe(NO_3_)_3_·9H_2_O, and Cu(NO_3_)_2_·H_2_O, respectively. Taking 0.5% Au-C_3_N_4_ as an example, 200 mg g-C_3_N_4_ was dispersed into 2 mL deionized water, then 200 μL HAuCl_4_·4H_2_O solution (10.4 mg/mL, containing 1 mg Au) was added into the slurry. The slurry was stirred to dry at room temperature and then calcined at 300 °C for 4 h. Before reaction, the catalyst was treated with 10% H_2_/Ar at 300 °C for 2 hours at a ramp rate of 10 °C/min.

### Catalysts characterization

X-ray Diffraction (XRD) analysis was carried out using a PANalytical X’Pert^3^ Powder X-ray powder diffractometer equipped with a Cu Kα radiation source, at a scan rate of 9 °/min, in Analytic Instrumentation Center of College of Chemistry and Molecular Engineering at Peking University. The accelerating voltage and current were 40 kV and 40 mA, respectively.

The surface area of the samples was examined using a Micrometer ASAP 2020 plus Accelerated Surface Area & Porosimetry Sstem in Analytic Instrumentation Center of College of Chemistry and Molecular Engineering at Peking University, by the low-temperature N_2_ adsorption-desorption method. The surface area was calculated by Brunauer-Emmett-Teller (BET) model.

Ultraviolet and visible (UV-vis) spectra were collected at room temperature with a Shimadzu UV3600 Plus UV-VIS-NIR Spectrophotometer in Analytic Instrumentation Center of College of Chemistry and Molecular Engineering at Peking University. A white standard of BaSO_4_ was used as a reference.

Fourier transform infrared spectroscopy (FTIR) test was performed using a ThermoFisher NICOLET iS10 Spectrometer by transmission mode. The g-C_3_N_4_ sample was physically mixed with standard KBr at a mass ratio of 1:20. The mixed powder was pressed into a transparent sheet and tested by the spectrometer.

X-ray photoelectron spectroscopy (XPS) was performed on an AXIS Supra photoelectron spectrometer (Kratos Analytical Ltd.) using monochromatized Al Kα radiation in Analytic Instrumentation Center of College of Chemistry and Molecular Engineering at Peking University.

Raman spectra was collected with a Thermo Fisher Scientific DXRxi Micro Raman imaging spectrometer, using 780 nm laser as excitation light and EMCCD detector. The laser power is 2–24 mW with exposure time of 0.2 s.

Elemental analysis of catalyst and polystyrene sample was performed by an Elementar Analysensysteme GmbH Vario MICRO CUBE Elemental Analyzer in Analytic Instrumentation Center of College of Chemistry and Molecular Engineering at Peking University.

### Catalytic performance evaluation

The catalytic reactions were carried out in a 100 mL stainless-steel autoclave with a sapphire window and magnetic stirring system (Beijing century Senlong Co., Ltd. LC100 reactor). In a typical procedure, 50 mg catalyst and 20 mg polystyrene were dispersed into 30 mL solvent. The autoclave was purged with pure O_2_ to desired pressure without replacing the atmosphere of air (0.79 bar of N_2_ was used as quantitative internal standard). The autoclave was heated to the designed temperature (about 30 min), then was kept at reaction temperature and irradiated by a 300 W Xenon lamp for different time.

Products of the catalytic reactions were analyzed by Agilent 7820 A Gas Chromatography System and Agilent 1200 Series Liquid Chromatography System. Gas components, such as CO_2_, O_2_, N_2_, CO, were separated by a Porapark Q column and a 5 A molecular sieve column, then detected by a TCD detector. The content of each gas component was calculated using N_2_ as internal standard (IS). Quantitative nitrobenzene was added as internal standard into the solution after reaction. Products in liquid phase were separated with a C18 column using acetonitrile/water gradient elution.

Specific calculation methods are as follows:

The peak area in the chromatogram conforms to a linear response relationship in a certain concentration range:

1 $$\frac{S{{\mbox{(A)}}}}{k\left({{{{{{{\rm{A}}}}}}}}\right)n\left({{{{{{{\rm{A}}}}}}}}\right)}=\frac{S{{\mbox{(B)}}}}{k{({\rm B})}n{{\mbox{(B)}}}}=\frac{S{{\mbox{(IS)}}}}{n{{\mbox{(IS)}}}}$$

S(A), k(A), and n(A) are respectively chromatographic peak area, relative correction factor to internal standard (IS), and amount of substance of component A. Therefore, the amount of substance is calculated by internal standard method (taking benzoic acid as an example):

2 $$n\left({{\mbox{benzoic acid}}}\right)=\frac{S\left({{{{{{{\rm{benzoic}}}}\; {{{\rm{acid}}}}}}}}\right)}{S\left({{{{{{{\rm{nitrobenzene}}}}}}}},\;{{{{{{{\rm{IS}}}}}}}}\right)}$$

Conversion and selectivity were calculated according to the number of moles of carbon in the corresponding substance. This conversion definition is different from general definition.

3 $$	{{{{{{{\rm{conversion}}}}}}}}=\\ 	\frac{7\times n\left({{\mbox{benzoic acid}}}\right)+8\times n\left({{\mbox{acetophenone}}}\right)+7\times n\left({{\mbox{benzaldehyde}}}\right)+n\left({{{\mbox{CO}}}}_{{{\mbox{x}}}}\right)}{8\times n\left({{{\mbox{C}}}}_{8}{{{\mbox{H}}}}_{8}{{\mbox{unit}}}\right)}\times 100\%$$

4 $$	{{{\mbox{selectivity}}}}\, {{{\mbox{to}}}}\, {{{\mbox{organics}}}}=\\ 	\frac{7\times n\left({{\mbox{benzoic acid}}}\right)+8\times n\left({{\mbox{acetophenone}}}\right)+7\times n\left({{\mbox{benzaldehyde}}}\right)}{7\times n\left({{\mbox{benzoic acid}}}\right)+8\times n\left({{\mbox{acetophenone}}}\right)+7\times n\left({{\mbox{benzaldehyde}}}\right)+n\left({{{{{{{{\rm{CO}}}}}}}}}_{{{{{{{{\rm{x}}}}}}}}}\right)}\times 100\%$$

5 $$	{{{{{{{\rm{selectivity}}}}}}}}\,{{{{{{{\rm{in}}}}}}}}\,{{{{{{{\rm{liquid}}}}}}}}\,{{{{{{{\rm{phase}}}}}}}}\,({{{{{{{\rm{benzoic}}}}}}}}\,{{{{{{{\rm{acid}}}}}}}})=\\ 	\frac{{{{{{{{\rm{7}}}}}}}}\,\times\,{{n}}({{{{{{{\rm{benzoic}}}}}}}}\,{{{{{{{\rm{acid}}}}}}}})} {{{{{{{{\rm{7}}}}}}}}\,\times\, {{{{{n}}}}}\,({{{{{{{\rm{benzoic}}}}}}}}\,{{{{{{{\rm{acid}}}}}}}})+{{{{{{{\rm{8}}}}}}}}\,\times\,{{n}}({{{{{{{\rm{acetophenone}}}}}}}})+{{{{{{{\rm{7}}}}}}}}\,\times\, {{n}}({{{{{{{\rm{benzaldehyde}}}}}}}})} \times {{{{{{{\rm{100}}}}}}}}\%$$

6 $${{{{{{{\rm{yield}}}}}}}}=\frac{7\,\times n\left({{{\mbox{benzoic acid}}}}\right)+8 \times n\left({{\mbox{acetophenone}}}\right)+7\,\times n\left({{\mbox{benzaldehyde}}}\right)}{n\left({{{{{{{{\rm{CH}}}}}}}}}_{2}{{{\mbox{ unit}}}} \,{{{\mbox{in}}}} \,{{{\mbox{polystyrene}}}}\right)}\,\times 100\%$$

^1^H Nuclear Magnetic Resonance (NMR) spectrum of the separated benzoic acid (Supplementary Fig. 17) was collected by Bruker AVANCE III 400 MHz in Analytic Instrumentation Center of College of Chemistry and Molecular Engineering at Peking University.

Gas Chromatography-Mass Spectrometry (GC-MS) experiments were conducted by an Agilent 7890 A Gas Chromatography System equipped with a HP-5 MS UI column, analyzed by an Agilent 5975 C inert XL EI/CI MSD with triple-axis Detector.

### Characterization of polymers

Polystyrene samples after different reaction time and conditions were prepared by the following method: the post-reaction mixture (solvent, dissolved small molecules, catalysts, unreacted polystyrene) was centrifuged. The precipitate (catalysts, unreacted polystyrene) was dissolved by sonication in dichloromethane. The dichloromethane mixture was centrifuged to obtain polystyrene solution and used catalysts. The solution was then evaporated to dryness by a rotary evaporator with a vacuum pump to obtain solid polystyrene. The solid was washed with ethanol and dried at 60 °C in vacuum overnight to yield polystyrene samples after reaction.

Transmission Infrared (IR) spectra were collected by a Bruker Tensor 27 Infrared Spectrometer in Analytic Instrumentation Center of College of Chemistry and Molecular Engineering at Peking University. Polystyrene sample was physically mixed with standard KBr at a mass ratio of 1:20. The mixed powder was pressed into a transparent sheet and tested by the spectrometer.

Weight-average molecular weight (*M*_w_) and polymer dispersity index (PDI) of polystyrene samples were analyzed by a PL-GPC220 Gel Permeation Chromatography system. GPC standard polystyrenes were used to calibrate molecular weight. 1,2,4-trichlorobenzene (TCB) was used as the mobile phase and sample signals were detected by a refractive index (RI) detector at 150 °C.

Oxygen contents of polystyrene samples were analyzed by an Elementar Analysensysteme GmbH Vario MICRO CUBE Elemental Analyzer in Analytic Instrumentation Center of College of Chemistry and Molecular Engineering at Peking University. The polystyrene samples were dried at 60 °C in vacuum before tests.

### Characterization of radical species

Electron paramagnetic resonance (EPR) spectra were collected at the X-band with a Magnettech MS-5000 EPR spectrometer with a 100 kHz magnetic at a microwave power of 6.31 mW to detect the oxygen radicals in the reaction system. Typically, 50 mg g-C_3_N_4_ catalyst, 20 mg polystyrene, DMPO (10 mmol/L) were dispersed into 2 mL methanol (to detect •O_2_^‒^) or water (to detect •OH) by sonication at atmospheric air. The mixture was sampled using a capillary tube and placed in an EPR tube for experiment and irradiated by a 300 W Xenon lamp.

## Supplementary information

Supplementary Information

## Data Availability

Source data are provided with this paper.

## Source data

Source Data
