# Supplementary material for: Catalytic oxidation of polystyrene to aromatic oxygenates over a graphitic carbon nitride catalyst
Source: Nat Commun. 2022 Aug 16;13:4809. doi: 10.1038/s41467-022-32510-x (PMC9381527; doi:10.1038/s41467-022-32510-x)
Supplement: Supplementary file 1 — Supplementary Information [file 41467_2022_32510_MOESM1_ESM.pdf]

Supplementary Information

Catalytic Oxidation of Polystyrene to Aromatic Oxygenates over Graphitic Carbon Nitride Catalyst

Ruochen Cao,[1] Mei-Qi Zhang, [1] Chaoquan Hu,[2, 3] Dequan Xiao[4], Meng Wang[1,]*, and Ding Ma[1,]*

[1]Beijing National Laboratory for Molecular Sciences, College of Chemistry and Molecular Engineering, Peking University, Beijing 100871, People's Republic of China

[2]State Key Laboratory of Multiphase Complex Systems, Institute of Process Engineering, Chinese Academy of Sciences, Beijing 100190, People's Republic of China

[3]Nanjing IPE Institute of Green Manufacturing Industry, Nanjing 211135, People's Republic of China

[4]Center for Integrative Materials Discovery, Department of Chemistry and Chemical and Biomedical Engineering, University of New Haven, West Haven, Connecticut 06516, United States

*Correspondence: m.wang@pku.edu.cn (M.W.), dma@pku.edu.cn (D.M.)

Inventory of Supplementary Information

Experimental Materials

Supplementary Figures and Tables

Supplementary Fig. 1 XRD profiles of fresh and used g-C_3N_4 catalyst

Supplementary Fig. 2 BET analysis of g-C_3N_4 catalyst

Supplementary Fig. 3 FTIR spectrum of g-C_3N_4 catalyst

Supplementary Fig. 4 UV-vis spectra and Tauc plot analysis of g-C3N4 catalysts

Supplementary Fig. 5 XPS spectra of g-C_3N_4 catalyst

Supplementary Fig. 6 Raman spectra of fresh and used g-C_3N_4 catalysts

Supplementary Fig. 7 Polystyrene oxidation reaction system setup

Supplementary Fig. 8 Supplementary Fig. 8 Catalytic performance of polystyrene photo-oxidation on g-C_3N_4 catalysts at different temperature

Supplementary Fig. 9 Catalytic performance of polystyrene photo-oxidation on g-C_3N_4 catalysts with different O_2 pressure

Supplementary Fig. 10 Stability test of recycled catalyst g-C_3N_4

Supplementary Fig. 11 Time-evolution of different reaction products with or without light irradiation

Supplementary Fig. 12 Liquid chromatograms of reaction solution compared with standard compounds

Supplementary Fig. 13 Gas Chromatography-Mass Spectrometry (GC-MS) analysis of liquid product in reaction solution

Supplementary Fig. 14 EPR spectra of g-C_3N_4 photocatalysis system using DMPO as spin trap

Supplementary Fig. 15 Catalytic performances of PS, dimmers and phenols under standard reaction conditions

Supplementary Fig.16 Molecular weight distribution of the polystyrene reactant and the recovered polystyrene after reactions measured by GPC

Supplementary Fig. 17 ^{1}H Nuclear Magnetic Resonance (NMR) spectrum of the benzoic acid separated by column chromatography

Supplementary Table 1 Catalytic performance of polystyrene photo-oxidation and blank experiments in different solvent

Supplementary Table 2 Catalytic performance of polystyrene photo-oxidation on different C_3N_4 based catalysts

Supplementary Table 3 Catalytic performance of different PS reagent and PS plastic commodity

Supplementary Table 4 Oxygen contents of polystyrene samples obtained by elemental analysis

Supplementary Table 5 Elemental compositions of the fresh and used g-C_3N_4 catalysts.

Supplementary Table 6 Weight-average molecular weight (M_w) and polymer dispersity index (PDI) of polystyrene reactant and recovered polystyrene after reactions obtained by GPC measurements

Supplementary Table 7 Catalytic performances of PS with different thermal pretreatment

Supplementary Table 8 Time-evolution of different products for polystyrene photocatalytic oxidation reaction

Supplementary Table 9 Catalytic performance of polystyrene photo-oxidation on g-C_3N_4 catalysts at different temperature

Supplementary Table 10 Catalytic performance of polystyrene photo-oxidation on g-C_3N_4 catalysts with different O_2 pressure

Supplementary Table 11 Stability test of recycled catalyst g-C_3N_4

Supplementary Table 12 Time-evolution of different reaction products of oxygen treated polystyrene (PS-O)

Experimental Materials

Catalysts or precursors: Urea (Beijing Tong Guang Fine Chemicals Company), ZnO (30±10 nm, Shanghai Aladdin Biochemical Technology Co., Ltd.), SiC (fine, 320 grit, Alfa Aesar (China) chemicals Co., Ltd.), TiO_2 (P25, powder, 99.5%, Evonik industries AG.), Na_2S (Shanghai Macklin Biochemical Co., Ltd.), $(NH_4)_2MoO_4 \cdot 4H_2O$ (Sinopharm Chemical Reagent Co., Ltd.), Zinc acetate($Zn(Ac)_2$, 9 Ding Chemistry Shanghai Technology Co., Ltd.), LiCl (Beijing Tong Guang Fine Chemicals Company), KCl (Beijing Tong Guang Fine Chemicals Company), $HAuCl_4 \cdot 4H_2O$ and $H_2PtCl_6 \cdot 6H_2O$ (Sinopharm Chemical Reagent Co., Ltd.), Fe, Cu, and Zn (nitrates, Beijing Tong Guang Fine Chemicals Company).

Polystyrene: reagent (M_w = 800 Da, 2500 Da, 12000 Da, GPC standard; M_w = 50 kDa, 110 kDa, Sigma-Aldrich (Shanghai) Co., Ltd.; syndiotactic polystyrene, Shanghai Macklin Biochemical Co., Ltd.), polystyrene cup (Xiamen Yongchengxin Plastic Co., Ltd.), expanded polystyrene pellet (Henan Jingying Water Treatment Materials Co., Ltd.).

Other organic substrates: benzoic acid, acetophenone, phenol, and nitrobenzene (Sinopharm Chemical Reagent Co., Ltd.); ethylbenzene and p-isopropylphenol (Shanghai Macklin Biochemical Co., Ltd.); benzaldehyde (Xilong Scientific Co., Ltd); 1,2-diphenylethane (Jingen Biotechnology (Beijing) Co., Ltd.), 1,2-diphenylethanone (Shanghai Bide Pharmaceutical Technology Co., Ltd.), 5,5-dimethyl-1-pyrroline N-oxide (DMPO) (Beijing Baoying Tonghui Biotechnology Co., Ltd.)

Solvent: acetonitrile, acetone, dichloromethane, and methanol (Tianjin Concord Technology Co., Ltd.); tetrahydrofuran (THF, Beijing Tong Guang Fine Chemicals Company), diphenyl ether (Ph_2O, Energy Chemistry, Anhui Zesheng Technology Co., Ltd.), dimethyl sulfoxide (DMSO, Shanghai Aladdin Biochemical Technology Co., Ltd.), deuterated NMR solvent (DMSO, acetonitrile, acetone, chloroform, etc., Cambridge Isotope Laboratories, Inc.).

Supplementary Figures and Tables

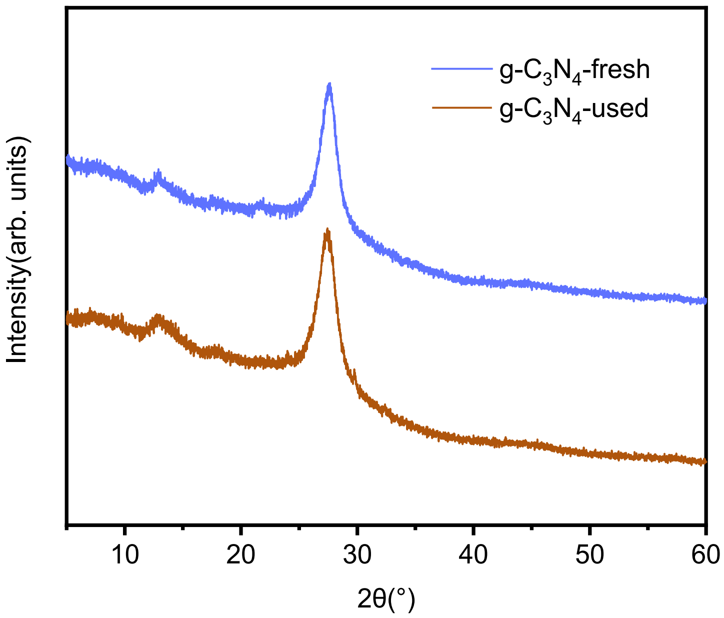

Supplementary Fig. 1 XRD profiles of fresh and used g-C_3N_4 catalyst. Peaks at 2θ of 13.1° and 27.5° correspond to in-planar repeated units and interplanar stacking of aromatic units, respectively.[1] Source data are provided as a Source Data file.

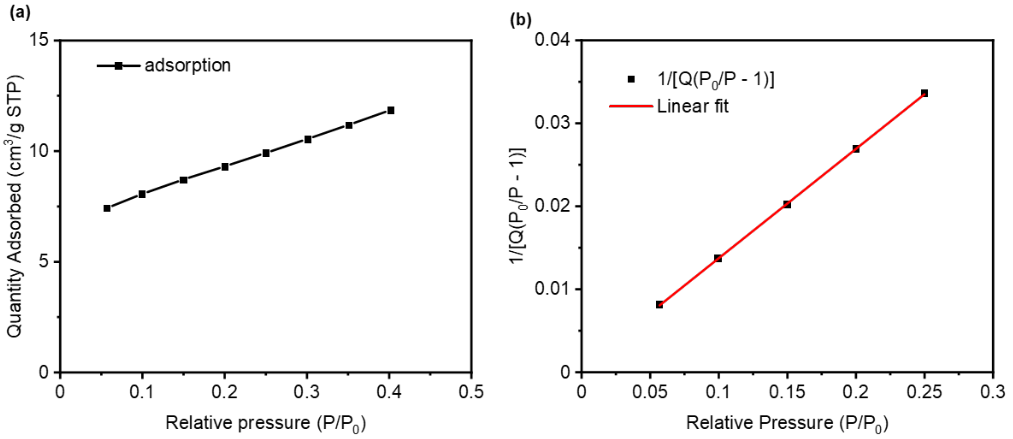

Supplementary Fig. 2 BET analysis of g-C_3N_4 catalyst. (a) Adsorption isotherm plot of g-C_3N_4;(b) BET surface area fitting plot of g-C_3N_4, slope = 0.1318 ± 0.0006 g/cm³ STP, y-intercept = 0.0006 ± 0.0001 g/cm³ STP, calculated surface BET area = 32.9 ± 0.2 m²/g. Source data are provided as a Source Data file.

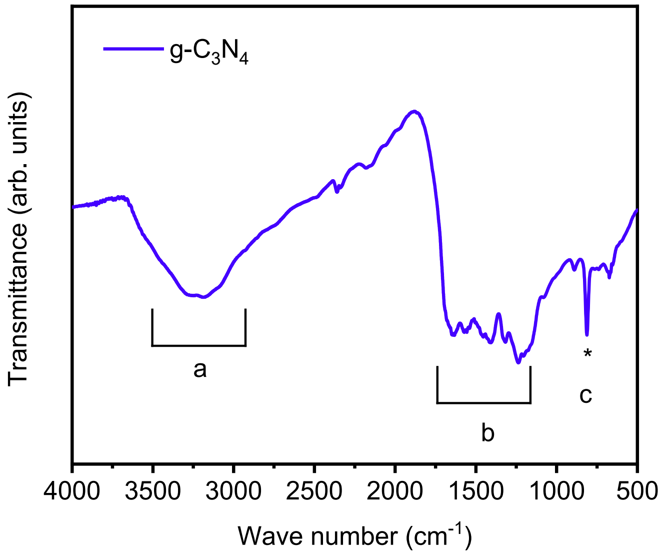

Supplementary Fig. 3 FTIR spectrum of g-C_3N_4 catalyst. Broad peaks in 3000 - 3500 cm^{-1} (a), multiple peaks in 1100 -1800 cm^{-1} (b) and sharp peak at 811 cm^{-1} (c) are respectively attributed to (a) stretching modes of -NH-, (b) stretching and bending modes of nitrogen heterocycles and (c) breathing mode of triazine unit.[2-4] Source data are provided as a Source Data file.

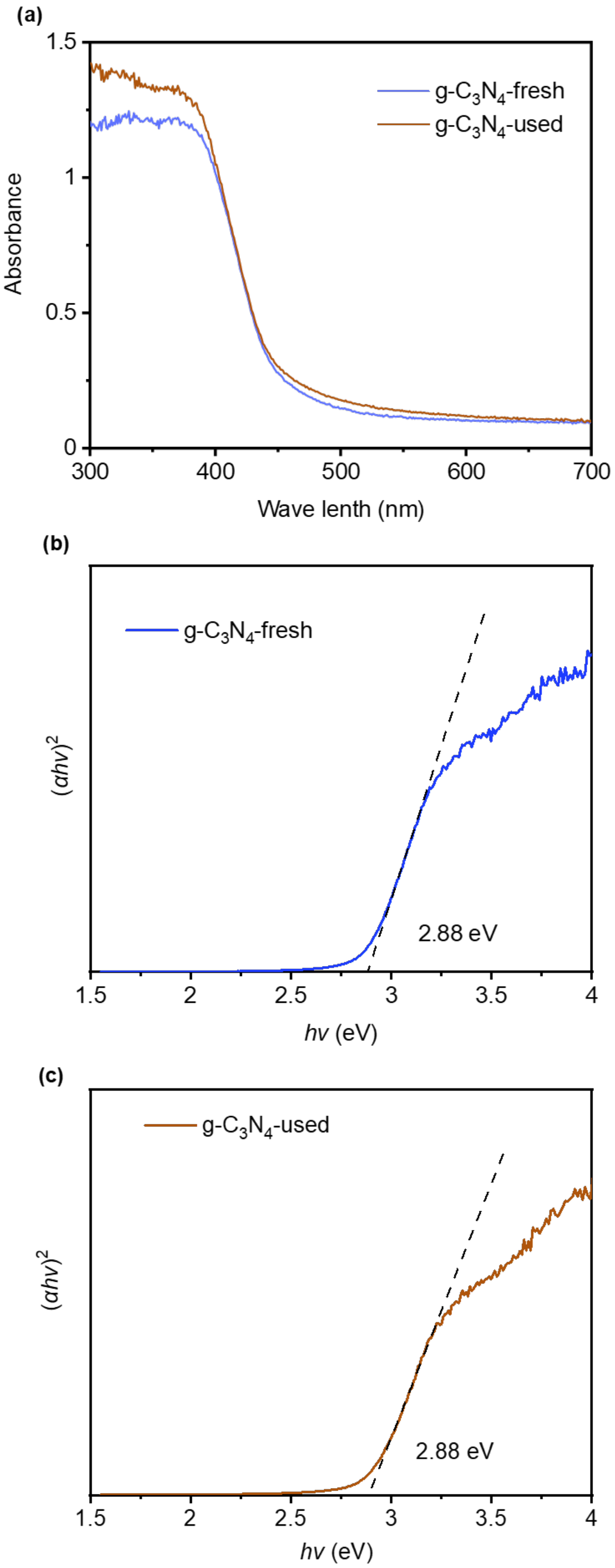

Supplementary Fig. 4 UV-vis spectra and Tauc plot analysis of g-C3N4 catalysts. (a) UV-vis spectra of fresh and used g-C_3N_4; (b)-(c) Tauc plot of (b) fresh g-C_3N_4 and (c) used g-C_3N_4 catalysts. Source data are provided as a Source Data file.

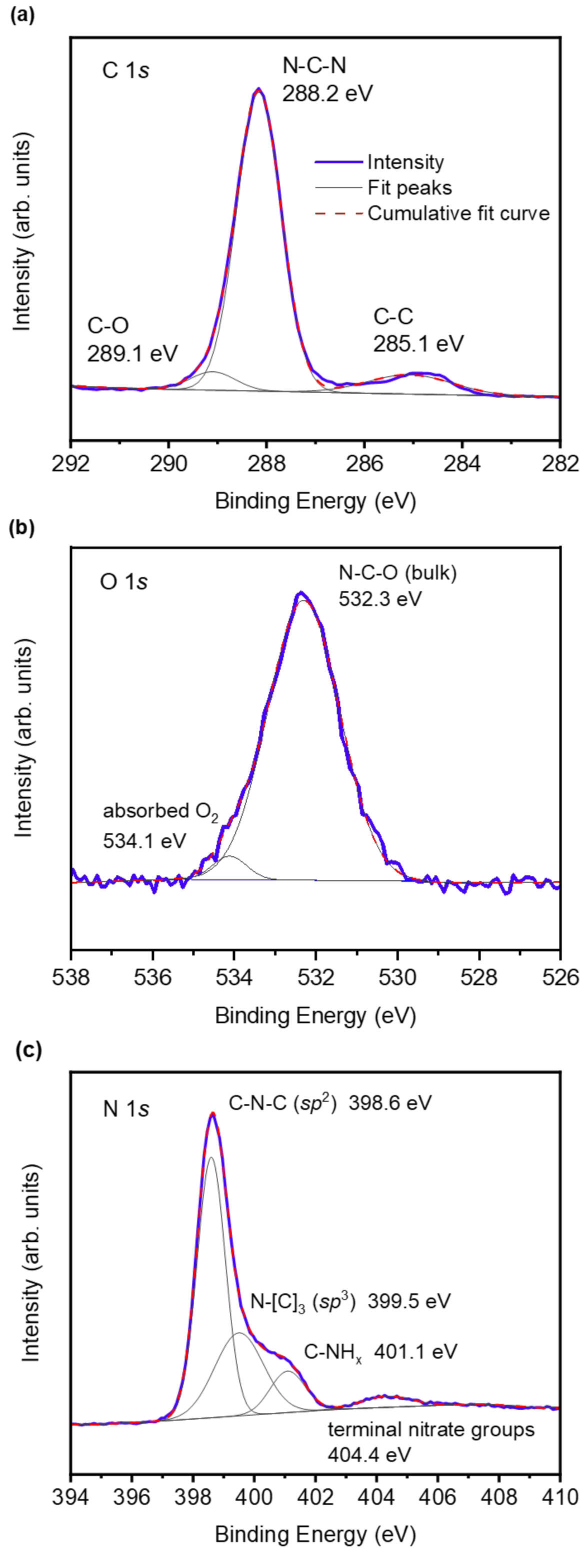

Supplementary Fig. 5 XPS spectra of g-C_3N_4 catalyst. (a) C 1*s* spectrum; (b) O 1*s* spectrum; (c) N 1*s* spectrum. The attribution of peaks refers to the previous literature[3,5,6]. Source data are provided as a Source Data file.

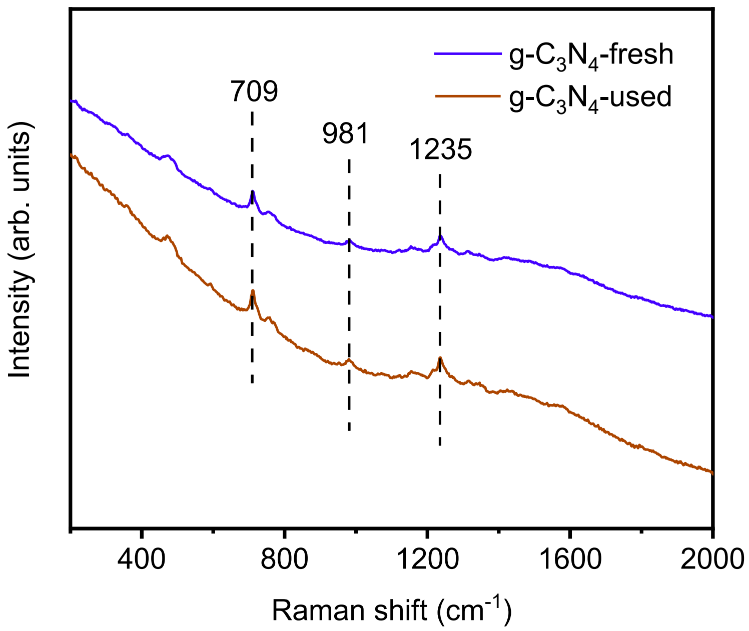

Supplementary Fig. 6 Raman spectra of fresh and used g-C_3N_4 catalysts. Raman shift peaks at 709 cm^{-1} and 981 cm^{-1} are attributed to different breathing modes of s-triazine[7], and peaks at 1235 cm^{-1} are attributed to stretching vibration modes of C=N and C–N in heterocycles[3]. Source data are provided as a Source Data file.

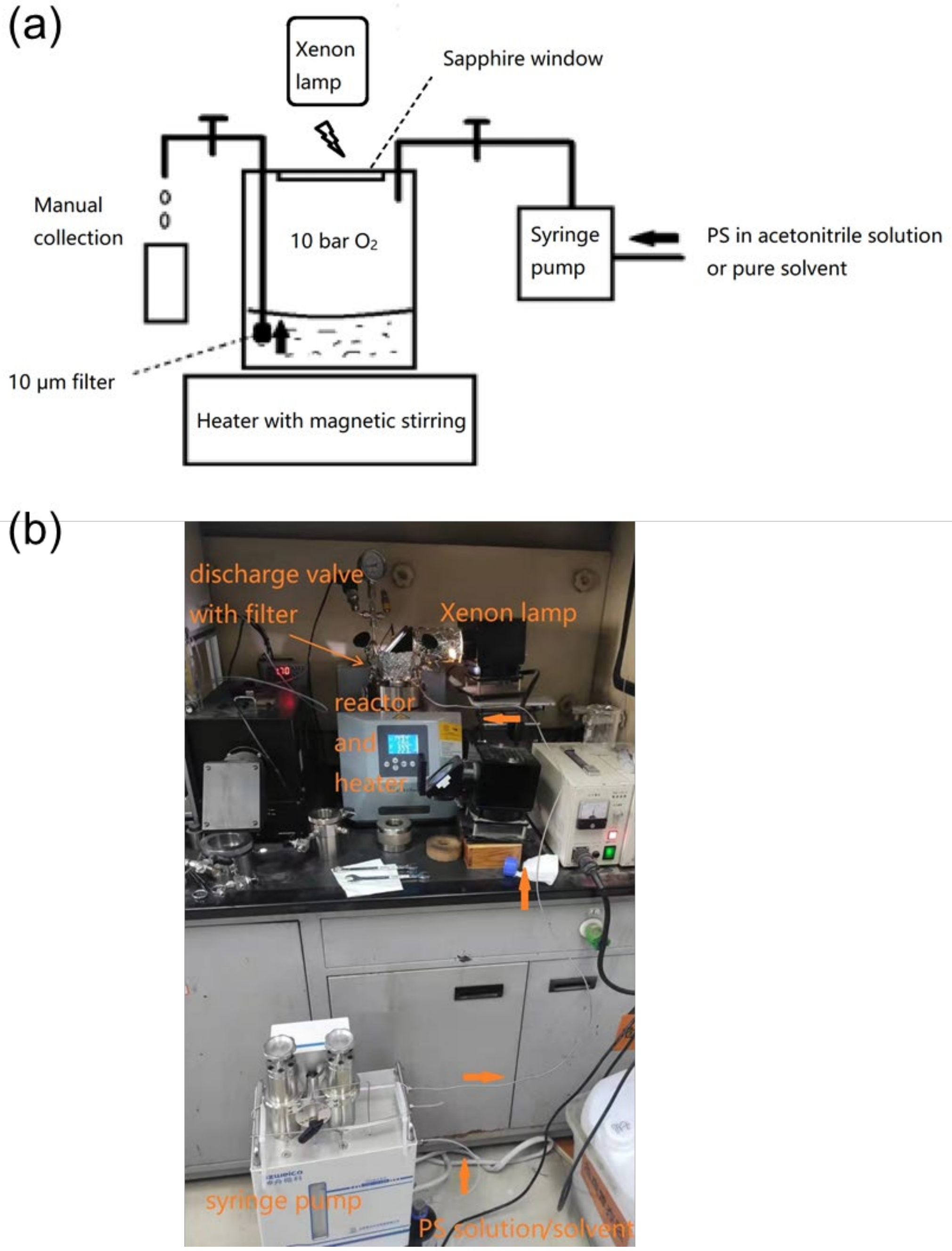

Supplementary Fig. 7 Polystyrene oxidation reaction system setup. (a) Schematic diagrams of polystyrene oxidation reaction system; (b) photos of reaction system. Note: pure solution was injected in flow reaction experiments and PS solution (~0.3 mg/mL) was injected in experiments with different WHSV.

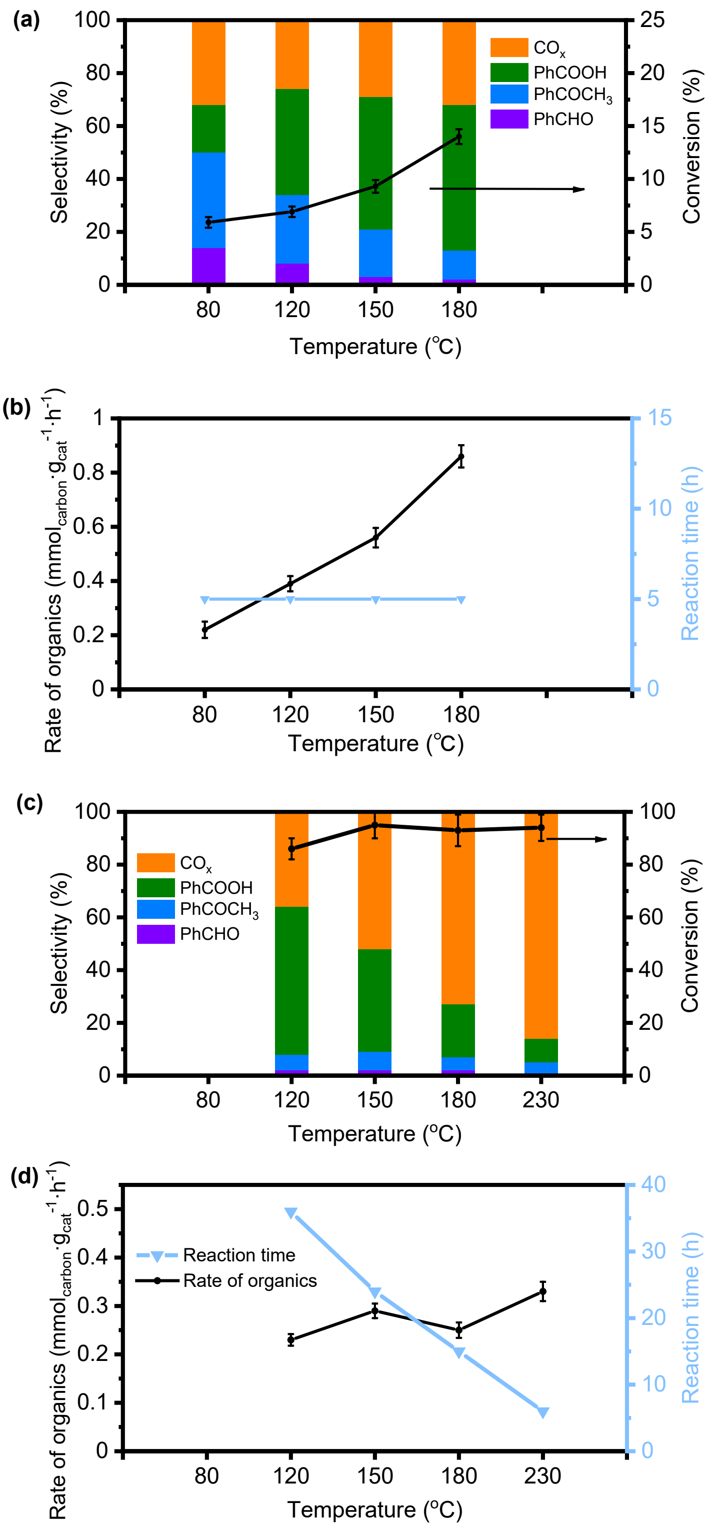

Supplementary Fig. 8 Catalytic performance of polystyrene photo-oxidation on g-C_3N_4 catalysts at different temperature. (a)-(b) Catalytic performance at low conversions and (c)-(d) catalytic performance at high conversions. Reaction conditions: 50 mg g-C_3N_4, 20 mg polystyrene (M_w ~ 50 kDa), 300 W Xenon lamp, 1 bar air, 30 mL acetonitrile solvent in 100 mL autoclave, 300 rpm magnetic stirring. The error bars represent the standard deviation of conversion in 3 parallel experiments. For detailed data, see Supplementary Table 9.

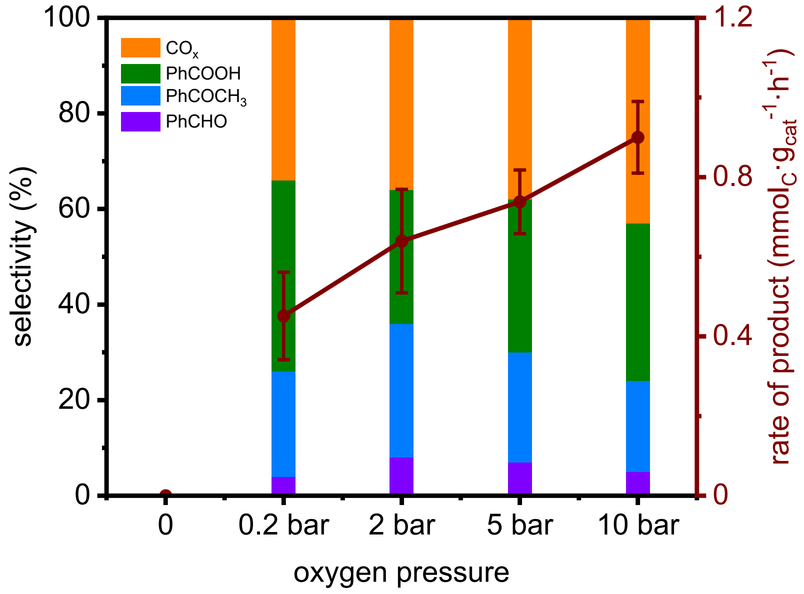

Supplementary Fig. 9 Catalytic performance of polystyrene photo-oxidation on g-C_3N_4 catalysts with different O_2 pressure. The blank control was carried out in nitrogen. Reaction conditions: 50 mg g-C_3N_4, 20 mg polystyrene (M_w ~ 50 kDa), 300 W Xenon lamp, 150 °C, 5 h, 30 mL acetonitrile solvent in 100 mL autoclave, 300 rpm magnetic stirring. The error bars represent the standard deviation of conversion in 3 parallel experiments. For detailed data, see Supplementary Table 10.

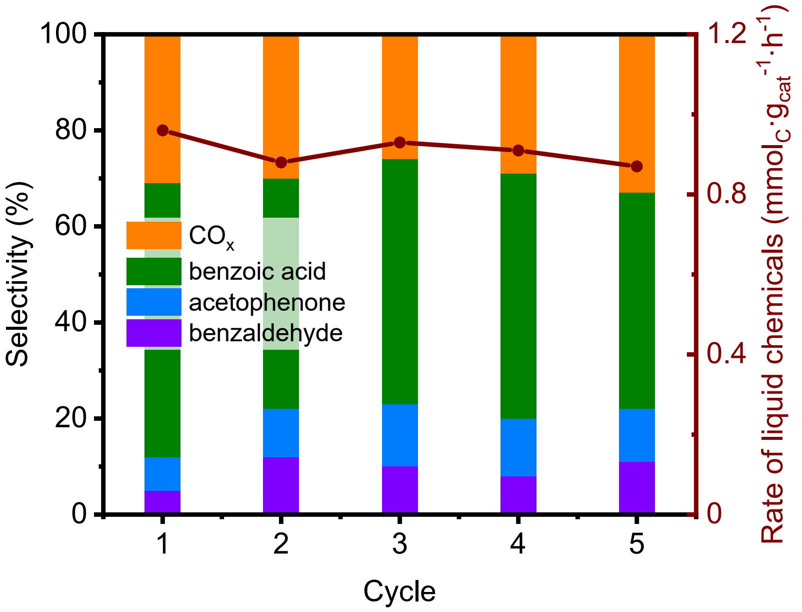

Supplementary Fig. 10 Stability test of recycled catalyst g-C_3N_4. Reaction conditions: 50 mg g-C_3N_4, 20 mg PS (M_w ~ 50 kDa), 30 mL acetonitrile, 150 °C, 10 bar O_2, 300W Xenon lamp, 5 h. After each cycle, PS and the solution was separated from the catalyst. The solution was separated by centrifugation and the solid was washed by CH_2Cl_2, in which PS can be dissolved but g-C_3N_4 remains. The remaining g-C_3N_4 was dried in air at 120 °C ant used for next cycle. Conversion of first cycle is 23%. Conversion of each cycle is proportional to product formation rate. For detailed data, see Supplementary Table 11.

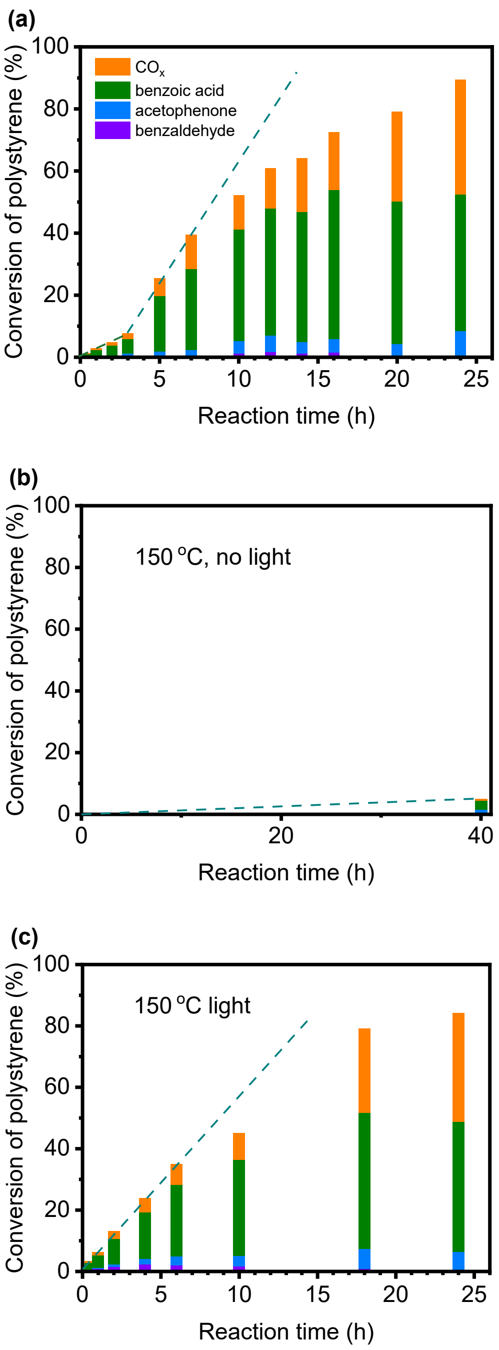

Supplementary Fig. 11 Time-evolution of different reaction products with or without light irradiation. (a) Time-evolution of different reaction products in 24 h at 150 ℃ of pristine polystyrene (see also figure 1a), other conditions: 50 mg g-C_3N_4, 10 mg polystyrene (M_w ~ 50 kDa), 300 W Xenon lamp, 30 mL acetonitrile solvent in 100 mL autoclave, 300 rpm magnetic stirring; (b) Reaction products in 40 h at 150 ℃ of pristine polystyrene without light irradiation; (c) Time-evolution of different reaction products in 24 h at 150 ℃ of oxygen treated polystyrene (PS-O), other conditions not mentioned in (b) and (c) are the same with (a). For detailed data, see Supplementary Table 12.

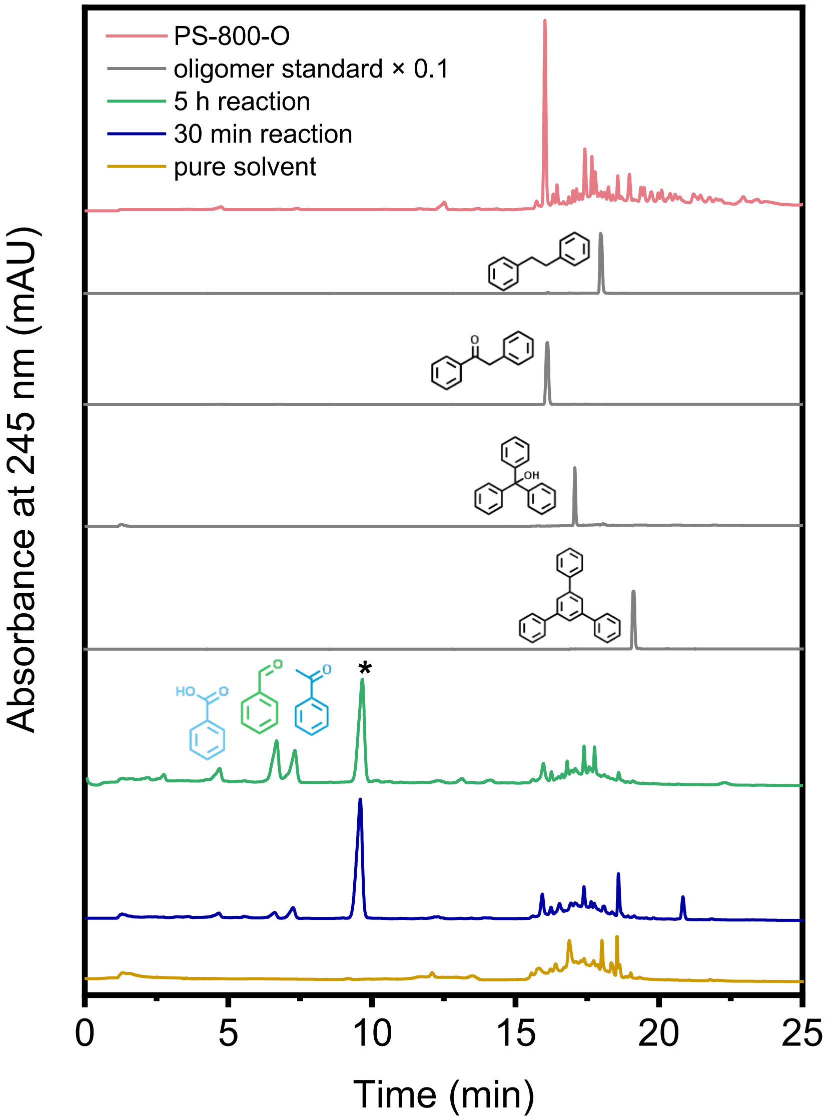

Supplementary Fig. 12 Liquid chromatograms of reaction solution compared with standard compounds. PS-800-O was obtained by treating the polystyrene with a M_w of 800 Da at 150 °C for 10 h, to simulate the mixture of polystyrene oligomers and their partial oxidation products. The signals of oligomers were reduced by a factor of ten. Asterisk* represent the signals of nitrobenzene (as internal standard). Source data are provided as a Source Data file.

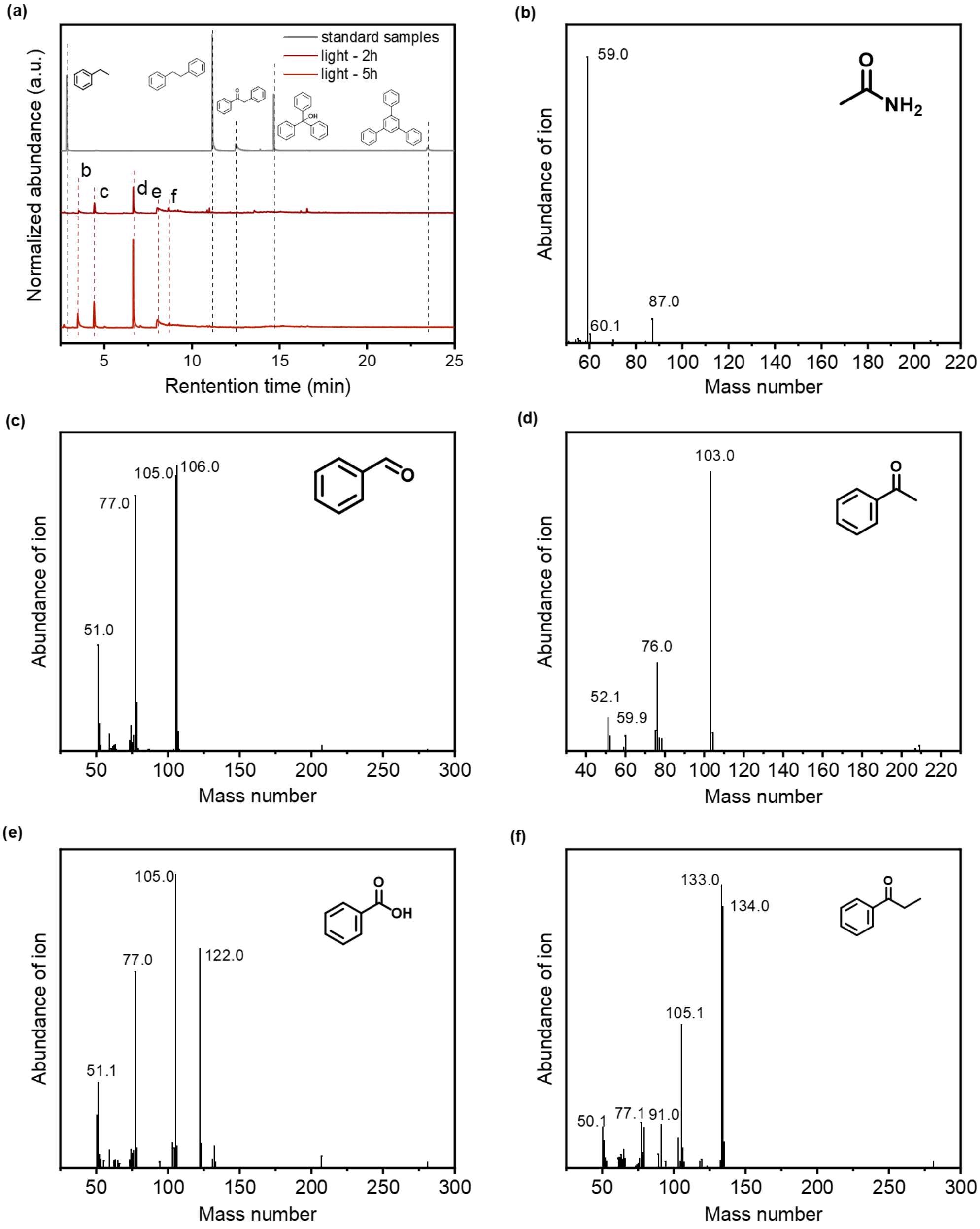

Supplementary Fig. 13 Gas Chromatography-Mass Spectrometry (GC-MS) analysis of liquid product in reaction solution. Reaction conditions: 150 °C under 10 bar O_2, 50 mg g-C_3N_4, 10 mg polystyrene (M_w ~ 50 kDa), 30 mL acetonitrile, 300 W Xenon lamp. (a) Gas chromatogram of solution after 2 h and 5 h reaction. (b) - (f) Mass Spectrometer results of peaks in GC. In the gas chromatogram of 2 h reaction, 10 - 20 min, there are some peaks that no reasonable result was matched by mass spectra. These signals might come from trace impurities or possible oligomeric intermediates in the system, but their content is much lower than that of products such as benzoic acid. Source data are provided as a Source Data file.

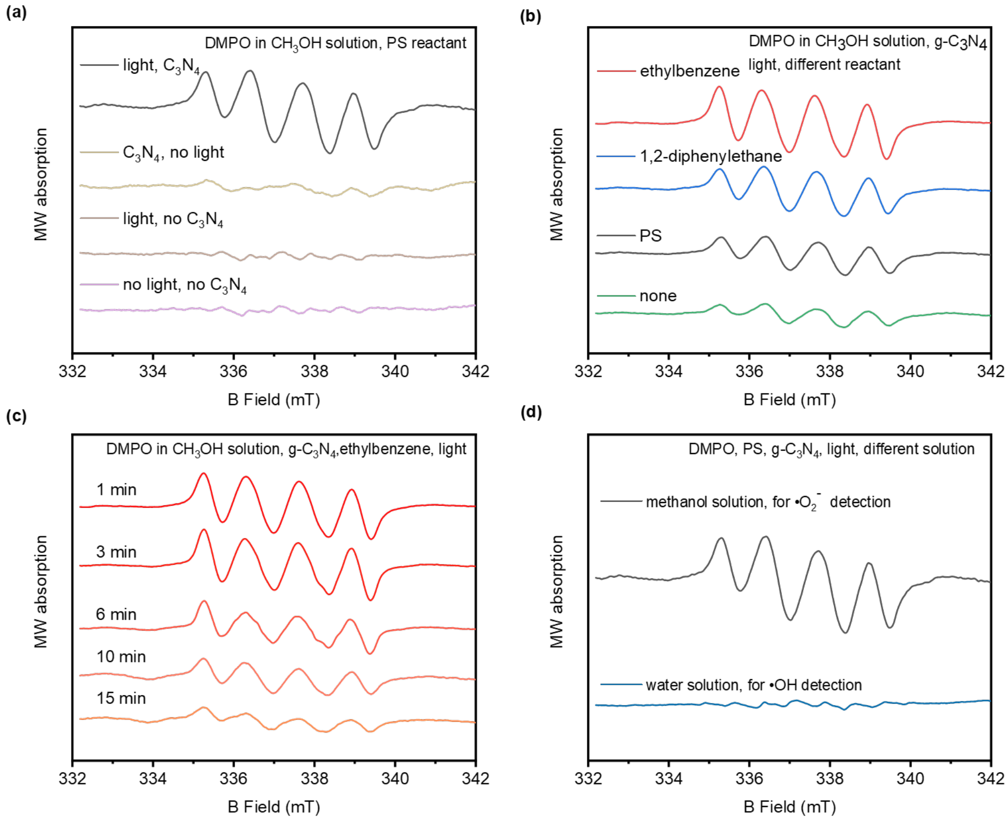

Supplementary Fig. 14 EPR spectra of g-C_3N_4 photocatalysis system using DMPO as spin trap. (a) EPR spectra under different illumination and catalyst conditions; (b) EPR spectra of g-C_3N_4 photocatalysis system in the presence of different reactant; (c) Time-dependent EPR spectra of g-C_3N_4 photocatalysis system using ethylbenzene as model reactant; (d) EPR spectra of g-C_3N_4 photocatalysis system in methanol and water. Source data are provided as a Source Data file.

Notes: EPR signal of DMPO-•O_2^- spin adduct show distorted quadruple peaks[4,8,9]. Positions and shapes of the peaks do not change with the change of reactants (b) and reaction time (c), indicating that no other radical species can be observed under the test conditions (room temperature, atmosphere air). There is no discernible signal in water solution (d), indicating •OH is not generated in g-C_3N_4 photocatalysis system.

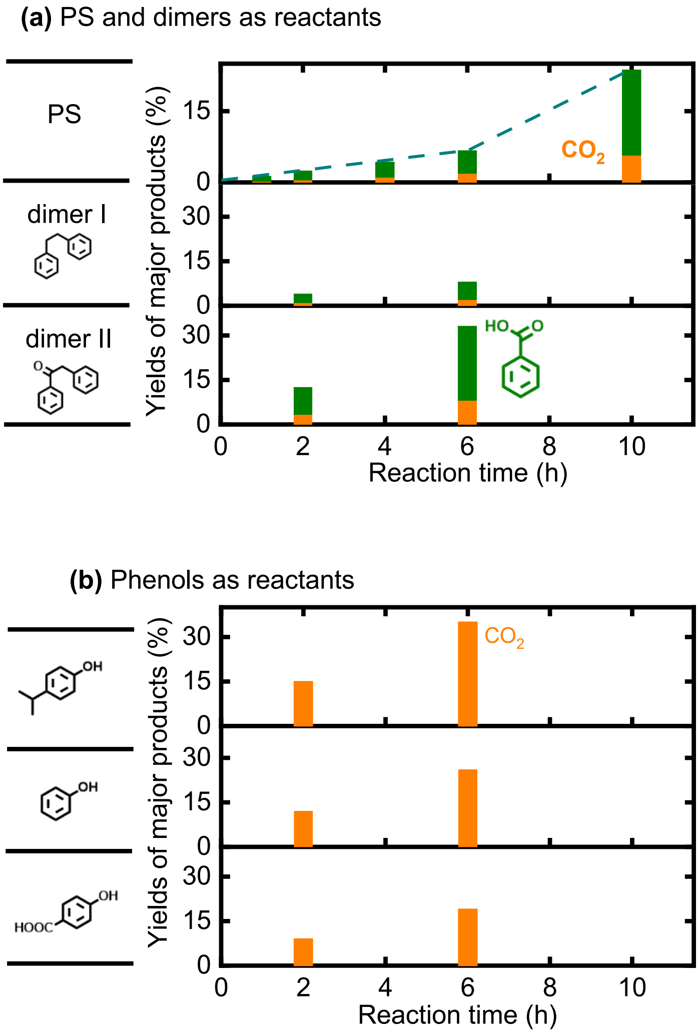

Supplementary Fig. 15 Catalytic performances of PS, dimmers and phenols under standard reaction conditions. (a)-(b) Product yield when using PS and dimers, and phenols as reactants under standard reaction conditions of 50 mg g-C_3N_4, 20 mg reactant, 150 °C, 300 W Xenon lamp, and 10 bar O_2. The reactants were rapidly converted into CO_2 without considerable amounts of organic products. Source data are provided as a Source Data file.

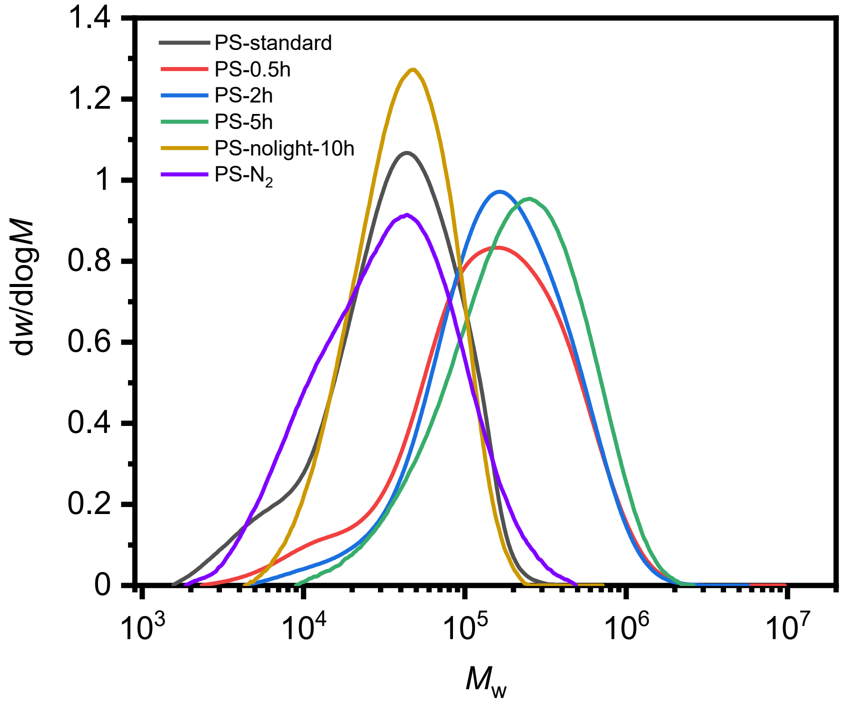

Supplementary Fig.16 Molecular weight distribution of the polystyrene reactant and the recovered polystyrene after reactions measured by GPC. Reaction conditions: 150 °C, 10 bar O_2, acetonitrile solvent, 300 W Xenon lamp with different reaction time in the presence of g-C_3N_4 catalyst. The PS-N_2 sample was treated under 10 bar N_2 for 10 h, other conditions are unchanged. Source data are provided as a Source Data file.

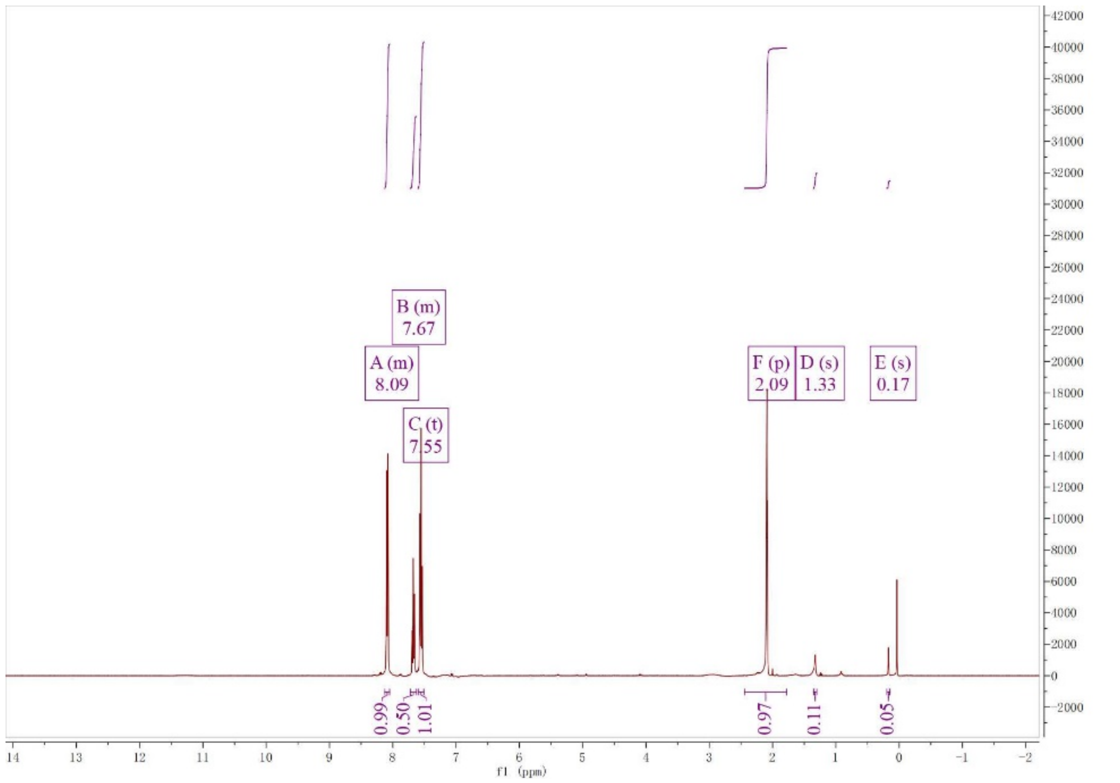

Supplementary Fig. 17 ^{1}H Nuclear Magnetic Resonance (NMR) spectrum of the benzoic acid separated by column chromatography. Peaks at 8.09, 7.67, and 7.55 ppm are the signals of benzoic acid. Peaks at chemical shift of 2.09 and 1.33 ppm correspond to deuterated acetone solvent and petroleum ether.

Supplementary Table 1 Catalytic performance of polystyrene photo-oxidation and blank experiments in different solvent. Other conditions: 50 mg catalyst, 80 °C, 30 mL solvent in 100 mL autoclave, 1 bar air, 300W Xenon lamp, 300 rpm magnetic stirring, 24 h.

solvent	catalyst	mass of PS/mg	rate of liquid product /(μmol$_c$·g_{cat}^{-1}·h^{-1})	rate of CO_2 evolution /(μmol$_c$·g_{cat}^{-1}·h^{-1})	rate of O_2 consumption /(μmol·g_{cat}^{-1}·h^{-1})	selectivity to organics [a] /%
THF	C_3N_4	20	0.023	0.18	0.25	11
THF	C_3N_4	0	0	0.15	0.21	0
acetone	C_3N_4	20	0.073	0.21	0.32	26
acetone	C_3N_4	0	0	0.14	0.19	0
acetonitrile	C_3N_4	20	0.23	0.15	0.33	61
acetonitrile	C_3N_4	0	0	0	0	0
acetonitrile [b]	C_3N_4	10	0.49	0.34	0.72	59
acetonitrile [b]	C_3N_4	0	0	0.008	0.018	0
acetonitrile [b]	none	0	0	0	0	0

[a] selectivity to organics = (*n*(carbon in benzoic acid, acetophenone and benzaldehyde)/(*n*(carbon in benzoic acid, acetophenone and benzaldehyde) + *n*($CO_{x\ total}$))) × 100%, CO_x from solvent oxidation is included.

[b] Optimized reaction condition: catalyst 50 mg, 150 °C, 10 bar O_2, 30 mL solvent in 100 mL autoclave, 300 rpm magnetic stirring, Xenon lamp, 24 h.

Supplementary Table 2 Catalytic performance of polystyrene photo-oxidation on different C_3N_4 based catalysts. Other conditions: 50 mg catalyst, 20 mg PS, 30 mL CH_3CN solvent in 100 mL autoclave, 1 bar air, 300W Xenon lamp, 300 rpm magnetic stirring, 12h.

catalyst	temperature/°C	selectivity to organics/%	conversion/%
C_3N_4	80	60	15
0.1%Au/C_3N_4	80	57	16
0.5%Au/C_3N_4	80	41	21
0.5%Pt/C_3N_4	80	46	19
0.5%Fe/C_3N_4	80	38	17
0.5%Cu/C_3N_4	80	53	17

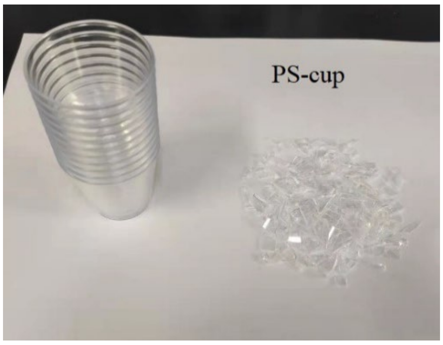

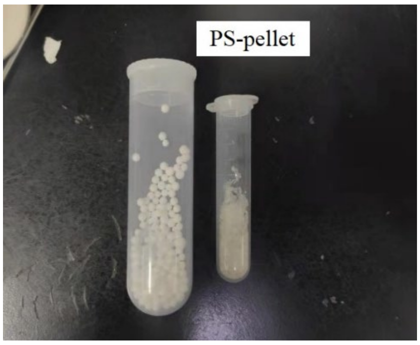

Supplementary Table 3 Catalytic performance of different PS reagent and PS plastic commodity. PS - 5 h refers to isolated PS from 5 h typical photo-oxidation reaction. Other conditions: g-C_3N_4 catalyst 50 mg, PS 20 mg, 150 ℃, 10 bar O_2, 30 mL CH_3CN solvent in 100 mL autoclave, 300 rpm magnetic stirring, Xenon lamp, 15 h. By extending reaction time to 60 h, the conversions of all PS samples are over 95%.

entry	PS type	conversion (%)	selectivity to organics (%)
1	PS - 800 Da	68	57
2	PS - 2500 Da	52	51
3	PS - 12 kDa	48	56
4	PS - 50 kDa	44	54
6	PS - 110 kDa	36	61
7	PS - cup	23	56
8	PS - pellet	48	63
9	syndiotactic PS	40	62
10 [a]	syndiotactic PS	95	46
11	PS - 5 h	50	58
12[a]	PS - 5 h	97	43

[a] The reaction time was prolonged to 60 h for full conversion. Other conditions are the same as entry 10.

Supplementary Table 4 Oxygen contents of polystyrene samples obtained by elemental analysis. The samples PS-10 h-thermal and PS-2 h-light were obtained by heating polystyrene in acetonitrile at 150 °C in the presence of g-C_3N_4 for corresponding time with and without light irradiation, respectively.

entry	PS sample	Oxygen mass content (%)
1	PS-50 kDa	0.13 ± 0.03
2	PS-10 h-thermal (PS-O)	0.35 ± 0.01
3	PS-2 h-light	0.47 ± 0.01

Supplementary Table 5 Elemental compositions of the fresh and used g-C_3N_4 catalysts.

catalyst		g-C_3N_4-fresh	g-C_3N_4-used
mass content (%)	C	34.1 ± 0.1	34.1 ± 0.1
	N	58.5 ± 0.1	58.4 ± 0.2
	H	5.4 ± 0.3	5.5 ± 0.2
	O	2.2 ± 0.1	2.2 ± 0.1
elemental proportion (%)	C	22.6 ± 0.1	22.5 ± 0.1
	N	33.3 ± 0.1	33.0 ± 0.1
	H	43.0 ± 0.8	43.5 ± 0.8
	O	1.1 ± 0.1	1.1 ± 0.1
C/N ratio		0.68	0.68

Supplementary Table 6 Weight-average molecular weight (M_w) and polymer dispersity index (PDI) of polystyrene reactant and recovered polystyrene after reactions obtained by GPC measurements. Standard reaction conditions: 50 mg g-C_3N_4, 20 mg polystyrene (M_w ~ 50 kDa), 30 mL acetonitrile, 300W Xenon lamp at 150°C, 10 bar O_2, in 100 mL autoclave, 300 rpm magnetic stirring.

entry	PS sample	M_w	PDI
1	PS-50 kDa	49753	2.33
2 [a]	PS-no light-10h	50597	1.60
3 [b]	PS-N_2	47198	2.51
4	PS-0.5 h	235460	3.49
5	PS-2 h	246025	2.54
6	PS-5 h	303100	2.41
7	PS-10 h	310112	2.15

[a] In this experiment, no lamp was used and the window on the reactor was blocked.

[b] This experiment was performed under 10 bar N_2 with heating at 150°C and Xenon lamp irradiation.

Supplementary Table 7 Catalytic performances of PS with different thermal pretreatment [a]

entry	pretreatment of PS	conversion (%)	selectivity of organics (%)
1	none (PS)	31	71
2	150°C, 10 bar O_2, 40 h (PS-O)	36	73
3	220°C, air, 5 h (PS-1)	58	82
4	300°C, air, 1 h (PS-2)	39	79
5	350°C, N_2, 1 h [b] (PS-3)	27	70

[a] reaction conditions: 50 mg g-C_3N_4, 20 mg PS, 30 mL CH_3CN in 100 mL autoclave, 300W Xenon lamp, 150°C, 10 bar O_2, 8h.

[b] the pretreated PS has a solubility of ~ 0.3 mg/mL in CH_3CN, 60 mL CH_3CN was used in entry 5.

Supplementary Table 8 Time-evolution of different products for polystyrene photocatalytic oxidation reaction, at 150 °C under 10 bar O_2, 50 mg g-C_3N_4, 10 mg polystyrene (M_w ~ 50 kDa), 30 mL acetonitrile, 300 W Xenon lamp. The errors represent standard deviation of conversion in 3 parallel experiments. This table is the data for plotting Figure 1a and Supplementary Figure 11a.

Reaction time / h	Yield [a] / % benzoic acid	acetophenone	benzaldehyde	CO_x	Conversion / %	Error of conversion /%
0	0	0	0	0	0	0
0.5	0	0	1.1	0.3	1.4	0.2
1	0	0.5	1.9	0.7	3.1	0.3
2	0	0.6	3.2	1.4	5.2	0.7
3	0	1.1	4.8	2.6	8.5	0.9
5	0.4	1.4	18	7.7	28	3
7	0.6	1.8	26	11	39	4
10	1.2	4	36	13	54	4
12	1.7	5.3	41	14.9	63	3
14	1.2	3.7	42	20.3	67	4
16	1.5	4.4	48	22.6	77	4
20	0	4.2	46	36	86	6
24	0	8.5	44	43	96	6
36	0	6.3	35	56.9	98	8

[a] yield was defined as follow, taking benzoic acid as example:

$$\text{yield(benzoic acid)} = \frac{7\times n(\text{benzoic acid})}{8\times n(\text{C}_8\text{H}_8\text{ unit})}\times 100\%$$

conversion = yield(benzoic acid) + yield(acetopehnone) + yield(benzaldehyde) + yield(CO_x)

Supplementary Table 9 Catalytic performance of polystyrene photo-oxidation on g-C_3N_4 catalysts at different temperature. Reaction conditions: 50 mg g-C_3N_4, 20 mg polystyrene (M_w ~ 50 kDa), 300 W Xenon lamp, 1 bar air, 30 mL acetonitrile solvent in 100 mL autoclave, 300 rpm magnetic stirring. This table is the data for plotting Supplementary Figure 8.

Temperature/ °C	Selectivity / %				Conversion / %	Rate of / $mmol_{carbon}\cdot g_{cat}^{-1}\cdot h^{-1}$	Reaction time / h
	benzoic acid	acetophenone	benzaldehyde	CO_x			
80	18	36	14	32	5.9 ± 0.5	0.22 ± 0.3	5
120	40	26	8	28	6.9 ± 0.5	0.39 ± 0.3	5
150	50	18	3	29	9.3 ± 0.6	0.56 ± 0.4	5
180	55	11	2	32	14 ± 0.7	0.86 ± 0.4	5
120	56	6	2	36	86 ± 4	0.23 ± 0.1	36
150	39	7	2	52	95 ± 5	0.29 ± 0.2	24
180	20	5	2	73	93 ± 6	0.25 ± 0.2	15
230	9	4	1	86	94 ± 5	0.33 ± 0.2	6

Supplementary Table 10 Catalytic performance of polystyrene photo-oxidation on g-C_3N_4 catalysts with different O_2 pressure. The blank control was carried out in nitrogen. Reaction conditions: 50 mg g-C_3N_4, 20 mg polystyrene (M_w ~ 50 kDa), 300 W Xenon lamp, 150 °C, 5 h, 30 mL acetonitrile solvent in 100 mL autoclave, 300 rpm magnetic stirring. This table is the data for plotting Supplementary Figure 9.

Oxygen pressure / bar	Selevtivity / %				Conversion / %	Rate of organics/ $mmol_{carbon}\cdot g_{cat}^{-1}\cdot h^{-1}$
	benzoic acid	acetophenone	benzaldehyde	CO_x		
0	0	0	0	0	0	0
0.2	40	22	4	34	11 ± 3	0.45 ± 0.11
2	28	28	8	42	15 ± 3	0.64 ± 0.13
5	32	23	7	38	19 ± 2	0.74 ± 0.08
10	33	19	5	43	22 ± 2	0.90 ± 0.09

Supplementary Table 11 Stability test of recycled catalyst g-C_3N_4. Reaction conditions: 50 mg g-C_3N_4, 20 mg PS (M_w ~ 50 kDa), 30 mL acetonitrile, 150 °C, 10 bar O_2, 300W Xenon lamp, 5 h. After each cycle, PS and the solution was separated from the catalyst. This table is the data for plotting Supplementary Figure 10.

Reaction cycle	Selectivity / %				Conversion / %	Rate of organics/ $mmol_{carbon}\cdot g_{cat}^{-1}\cdot h^{-1}$
	benzoic acid	acetophenone	benzaldehyde	CO_x		
1	57	7	5	31	23	0.96
2	48	10	12	30	21	0.88
3	51	13	10	26	22	0.93
4	51	12	8	29	22	0.91
5	45	11	11	33	21	0.87

Supplementary Table 12 Time-evolution of different reaction products of oxygen treated polystyrene (PS-O). Reaction conditions: 50 mg g-C_3N_4, 10 mg PS-O, 300 W Xenon lamp, 30 mL acetonitrile solvent in 100 mL autoclave, 300 rpm magnetic stirring. The first entry shows the 40 h reaction without light of pristine polystyrene at 150 °C. This table is the data for plotting Supplementary Figure 11b-c.

Reaction time / h	Yield [a] / %				Conversion / %
	benzoic acid	acetophenone	benzaldehyde	CO_x	
40 [b]	2.9	1.1	0.3	0.6	4.9
0.5	2.2	0.1	0.5	0.6	3.4
1	4.2	0.3	0.8	1.1	6.4
2	8.2	0.9	1.5	2.5	13
4	15	1.9	2.3	4.6	24
6	23	2.8	2.1	6.7	35
10	31	3.4	1.6	8.7	45
18	44	6.4	0.9	27	79
24	42	5.9	0.5	35	84

[a] yield was defined as follow, taking benzoic acid as example:

$$\text{yield(benzoic acid)} = \frac{7\times n(\text{benzoic acid})}{8\times n(\text{C}_8\text{H}_8\text{ unit})}\times 100\%$$

conversion = yield(benzoic acid) + yield(acetophenone) + yield(benzaldehyde) + yield(CO_x)

[b] PS (M_w ~ 50 kDa), no light irradiation.

Supplementary Reference

1 Wang, X. *et al.* A metal-free polymeric photocatalyst for hydrogen production from water under visible light. *Nature Materials* **8**, 76-80 (2009).

2 Han, Q., Wang, B., Zhao, Y., Hu, C. & Qu, L. A Graphitic-C_3N_4 “Seaweed” Architecture for Enhanced Hydrogen Evolution. *Angewandte Chemie International Edition* **54**, 11433-11437 (2015).

3 Yuan, Y. *et al.* High-yield synthesis and optical properties of g-C_3N_4. *Nanoscale* **7**, 12343-12350 (2015).

4 Zhao, H. *et al.* Photo-assisted separation of noble-metal-free oxidation and reduction cocatalysts for graphitic carbon nitride nanosheets with efficient photocatalytic hydrogen evolution. *Applied Catalysis B: Environmental* **280**, 119456 (2021).

5 Martin, D. J. *et al.* Highly Efficient Photocatalytic H_2 Evolution from Water using Visible Light and Structure-Controlled Graphitic Carbon Nitride. *Angewandte Chemie International Edition* **53**, 9240-9245 (2014).

6 Thomas, A. *et al.* Graphitic carbon nitride materials: variation of structure and morphology and their use as metal-free catalysts. *Journal of Materials Chemistry* **18**, 4893-4908 (2008).

7 Tonda, S., Kumar, S., Kandula, S. & Shanker, V. Fe-doped and -mediated graphitic carbon nitride nanosheets for enhanced photocatalytic performance under natural sunlight. *Journal of Materials Chemistry A* **2**, 6772-6780 (2014).

8 Long, B., Ding, Z. & Wang, X. Carbon Nitride for the Selective Oxidation of Aromatic Alcohols in Water under Visible Light. *ChemSusChem* **6**, 2074-2078 (2013).

9 Corp, K. L. & Schlenker, C. W. Ultrafast Spectroscopy Reveals Electron-Transfer Cascade That Improves Hydrogen Evolution with Carbon Nitride Photocatalysts. *Journal of the American Chemical Society* **139**, 7904-7912 (2017).
